# *FLOURY ENDOSPERM7* encodes a regulator of starch synthesis and amyloplast development essential for peripheral endosperm development in rice

**DOI:** 10.1093/jxb/erv469

**Published:** 2015-11-24

**Authors:** Long Zhang, Yulong Ren, Bingyue Lu, Chunyan Yang, Zhiming Feng, Zhou Liu, Jun Chen, Weiwei Ma, Ying Wang, Xiaowen Yu, Yunlong Wang, Wenwei Zhang, Yihua Wang, Shijia Liu, Fuqing Wu, Xin Zhang, Xiuping Guo, Yiqun Bao, Ling Jiang, Jianmin Wan

**Affiliations:** ^1^State Key Laboratory for Crop Genetics and Germplasm Enhancement, Jiangsu Plant Gene Engineering Research Center, Nanjing Agricultural University, Nanjing 210095, PR China; ^2^National Key Facility for Crop Resources and Genetic Improvement, Institute of Crop Science, Chinese Academy of Agricultural Sciences, Beijing 100081, PR China; ^3^College of Life Sciences, Nanjing Agricultural University, Nanjing 210095, PR China

**Keywords:** Amyloplast, DUF1338, endosperm cells, *FLOURY ENDOSPERM7*, rice (*Oryza sativa*), starch synthesis.

## Abstract

In cereal crops, starch synthesis and storage depend mainly on a specialized class of plastids, termed amyloplasts. Despite the importance of starch, the molecular machinery regulating starch synthesis and amyloplast development remains largely unknown. Here, we report the characterization of the rice (*Oryza sativa*) *floury endosperm7* (*flo7*) mutant, which develops a floury-white endosperm only in the periphery and not in the inner portion. Consistent with the phenotypic alternation in *flo7* endosperm, the *flo7* mutant had reduced amylose content and seriously disrupted amylopectin structure only in the peripheral endosperm. Notably, *flo7* peripheral endosperm cells showed obvious defects in compound starch grain development. Map-based cloning of *FLO7* revealed that it encodes a protein of unknown function. FLO7 harbors an N-terminal transit peptide capable of targeting functional FLO7 fused to green fluorescent protein to amyloplast stroma in developing endosperm cells, and a domain of unknown function 1338 (DUF1338) that is highly conserved in green plants. Furthermore, our combined β-glucuronidase activity and RNA *in situ* hybridization assays showed that the *FLO7* gene was expressed ubiquitously but exhibited a specific expression in the endosperm periphery. Moreover, a set of *in vivo* experiments demonstrated that the missing 32 aa in the *flo7* mutant protein are essential for the stable accumulation of FLO7 in the endosperm. Together, our findings identify FLO7 as a unique plant regulator required for starch synthesis and amyloplast development within the peripheral endosperm and provide new insights into the spatial regulation of endosperm development in rice.

## Introduction

Plastids, which are specific to plant cells, exist mainly in three forms—chloroplast, chromoplast, and leucoplast—depending on the developmental stage and cell type during plant life cycle. Among these, chloroplasts are specialized for conducting photosynthesis in green tissues, while amyloplasts (a type of leucoplasts) serve as the organelle for bulk synthesis and storage of starch in tubers, cotyledons, and endosperms (Lopez-Juez and Pyke, 2005; Sakamoto *et al.*, 2008). Starch is the most common and vitally important carbohydrate component in human diets and also has great value in industrial applications. However, the molecular and cellular machinery involved in regulating starch synthesis and amyloplast development remains poorly understood compared with the marked progress in the elucidation of chloroplast development over the last few decades (James *et al.*, 2003; Waters and Langdale, 2009).

Cereal crops accumulate large amounts of starch in seed endosperm as an energy reserve for supporting embryo development and germination. Starch, accounting for more than 70% of the seed weight, is a mixture of two homopolymers, amylose and amylopectin. Amylose is a linear polymer made of d-glucose molecules joined to each other via α-1,4 linkages. In contrast, the more abundant amylopectin is a highly branched polymer of d-glucose, where branching takes place through α-1,6 linkages (Hannah and James, 2008). Starch synthesis in cereal endosperm is a unique but relatively simple process of enzymatic reactions involved a few starch biosynthetic isozymes. Briefly, the synthesis of starch in plants begins with the enzyme ADP-glucose pyrophosphorylase (AGPase), which catalyzes the reaction of glucose 1-phosphate with ATP to produce the activated glucosyl donor ADP-glucose (ADPG). ADPGs are then used as substrates for both amylose-specific starch synthase (granule-bound starch synthase, GBSS) and amylopectin unique starch synthases (SSI, SSII, SSIII, and SSIV) to initiate amylose and amylopectin synthesis and elongation, respectively. For amylopectin synthesis, two additional classes of branching enzymes (BEI and BEII) are required to introduce α-1,6 branch points, while another two types of debranching enzymes [isoamylase (ISA) and pullulanase] most likely function in removing misplaced branch points to promote the crystallization of amylopectin (James *et al.*, 2003; Jeon *et al.*, 2010).

Rice (*Oryza sativa*), a primary food for more than half of the world’s population, has been a preferred model crop for molecular genetic and functional studies. Forward genetics approaches have been adopted to dissect the biological roles of the isozymes in starch synthesis in rice. For example, a weak mutation of rice AGPase large subunit *OsAPL2* accumulates small and round starch grains (Zhang *et al.*, 2012a). A loss-of-function mutation of *GBSSI* produces a waxy endosperm composed of amylose-free starch grains, confirming the essential role of *GBSSI* in amylose synthesis (Zhang *et al.*, 2012b). A deficiency of *SSIIIa* influences the structure of amylopectin, amylose content, and the physico-chemical properties of starch grains (Fujita *et al.*, 2007; Ryoo *et al.*, 2007). Additionally, mutations of the starch branching enzyme gene *BEIIb* and the starch debranching enzyme gene *ISA1* also lead to seriously disrupted amylopectin structure (Kubo *et al.*, 1999; Nishi *et al.*, 2001). Interestingly, a conspicuous phenotype for these mutants is the formation of opaque (originally used to characterize the starchy endosperm mutants in maize, such as *opaque1* [*o1*], *o2*, and *o5*; Wang *et al.*, 2012) and/or shrunken endosperms as compared with wild-type translucent and plumped grains, although they are distinguished from each other in various degree of chalkiness and/or shrinkage. Thus, it is likely that the loss-of-function of genes indirectly involved in starch synthesis can also cause an abnormal endosperm appearance. Indeed, such genes have been genetically identified and characterized. For example, the *FLO2* gene, encoding a nuclear-localized TPR-binding protein, has been shown to influence starch synthesis, potentially via interaction with transcription factors such as bHLHs to positively regulate expression of starch synthesis-associated genes (She *et al.*, 2010). *OsbZIP58* encodes a key transcriptional regulator required for starch synthesis through directly binding to the promoters of *OsAGPL3*, *GBSSI*, *OsSSIIa*, *SBE1*, *OsBEIIb*, and *ISA2* to promote their expression (Wang *et al.*, 2013). *Rice Starch Regulator 1* (*RSR1*), encoding a rice AP2/EREBP family transcription factor, is involved in the negative regulation of starch synthesis-related gene expression and consequently determines the amylose content and the fine structure of amylopectin (Fu and Xue, 2010). In contrast to these positive and negative regulators controlling starch biosynthesis gene expression, FLO6 (a CBM domain containing protein) modulates starch synthesis and compound starch grain formation through its interaction with ISA1 (Peng *et al.*, 2014).

In rice endosperm cells, each amyloplast forms a compound starch grain, composed of several dozen polyhedral, sharp-edged, and easily separable starch granules, which differ from single starch grains in other crops (Jane *et al.*, 1994; Yun and Kawagoe, 2010). Several mutants with altered starch grain morphology and size have been identified and characterized, for example, *ssg1/ssg2/ssg3*, allelic to the *ae* mutant, have increased numbers of small starch grains, accompanied by a floury endosperm appearance (Matsushima *et al.*, 2010). *SSG4* encodes a novel protein essential for controlling the size of starch grains; its mutation causes enlarged starch grains (Matsushima *et al.*, 2014). These significant advances have greatly promoted our understanding of the molecular mechanisms regulating starch synthesis and amyloplast development in rice endosperm; however, it is worth noting that mutants of these regulatory factors usually produce a white-core endosperm, indicating that they might play special roles in the development of the endosperm interior. Therefore, this raises a question of whether, irrespective of programmed cell death, there is special molecular machinery for the development of peripheral regions of endosperm.

Domains of unknown function (DUFs) represent a large set of uncharacterized protein families (Bateman *et al.*, 2010). Most DUFs are highly conserved in plants, indicating their important roles in plant growth and development. Growing evidence shows that some DUFs play important roles in plastid development. For instance, *YLC1*, a gene belonging to the DUF3353 superfamily, has been proved to play an important role in early leaf development in rice (Zhou *et al.*, 2013b). SSG4, a member of DUF490 superfamily, functions in regulating the size of chloroplasts and amyloplasts; its mutation also causes a leaf color phenotype (Matsushima *et al.*, 2014). However, DUFs required for amyloplast development, particularly in the endosperm, remain to be identified.

In this study, we identified a loss-of-function rice endosperm mutant, *flo7*, which displays an opaque-periphery endosperm phenotype. Map-based cloning revealed that *FLO7* encodes a domain of unknown function, DUF1338, containing a green-plant-unique protein, which is localized to the amyloplast stroma in developing endosperm cells. Our data suggest that FLO7 acts as a novel regulatory factor influencing peripheral development of endosperm via its unique endosperm expression, and our findings shed some light on the spatial regulation of endosperm development in rice.

## Materials and methods

### Plant materials and growth conditions

The *flo7* mutant was initially identified from a screening of about 10 000 T-DNA insertion M_2_ lines of *japonica* rice variety Nipponbare. The *flo7* mutant phenotype did not co-segregate with the T-DNA insertion site. Thus, the *flo7* mutant might be regenerated from an *in vitro* tissue culture. An F_2_ population was produced from *flo7* and an *indica* variety Pei’ai64 for mapping. All plants were grown in a paddy field at Nanjing Agricultural University during the natural growing season, and the developing seeds of the wild type (Nipponbare) and *flo7* at 4–21 d after fertilization (DAF) were used in the experiments.

### Microscopy

Scanning electron microscopy was performed as described previously (Kang *et al.*, 2005). Samples were examined with a HITACHI S-3400N scanning electron microscope (http://www.hitachi-hitec.com).

For the observation of endosperm cells, transverse sections of developing endosperms (approximately 1mm in thickness) of the wild type and *flo7* were fixed overnight in 0.1M phosphate buffer (pH 7.2) with 2% (v/v) glutaraldehyde and 2% (w/v) paraformaldehyde. After dehydration in an ethanol series, the samples were embedded in LR White resin (London Resin, Berkshire, UK, http://www.2spi.com), followed by sectioning with an ultramicrotome (Leica UC7; http://www.leicamicrosystems.com). Semi-thin sections (1 μm in thickness) were stained with 0.01 (v/v) toluidine blue for 10min and subsequently examined under a light microscope (80i; Nikon, http://www.nikon.com). Quantification of amyloplast numbers was done using ImageJ 1.46r software (http://rsbweb.nih.gov/ij).

For the ultrastructure observation of chloroplasts and amyloplasts, the leaves of 2-week-old seedlings and the developing seeds (4–12 DAF) were fixed for over 12h in 2.5% glutaraldehyde buffered with 0.2M phosphate buffer (pH 7.2). All sections were treated as described by Takemoto *et al.* (2002) and sectioned using an ultramicrotome (Power Tome-XL; RMC, http://www.rmcproducts.com). Microscopic observation was performed using a transmission electron microscope (H-7650; Hitachi, http://www.hitachi.com). To determine the filling ratio of amyloplasts in the outer endosperm cells, the amyloplast area and starch granule area were measured separately with ImageJ 1.46r. The filling ratio of amyloplasts (%) was calculated as the sum of starch granule areas in each amyloplast /area of each amyloplast×100.

### Characterization of amylose content and amylopectin structure

Separation of interior from exterior endosperm layers of mature endosperm was performed as described previously (Seguchi *et al.*, 2003). Briefly, rice grains were processed using a dehuller, and embryos were removed artificially with a knife. *flo7* brown rice was further processed using a rice polisher (Kett, Tokyo) until it displayed a transparent appearance. Fine flour was collected as the exterior endosperm layers of *flo7* mature endosperms, while the rest of the polished endosperm was further ground into fine flour as inner endosperm samples of *flo7* mature endosperm. Wild-type interior and exterior endosperm samples were collected separately with the same polish time as the *flo7* mutant. Amylose content was measured following the method described by Liu *et al.* (2009), and the chain length distribution of amylopectin was subsequently measured according to a previous report (Han *et al.*, 2012).

### Map-based cloning of the *FLO7* gene

To map the *FLO7* locus, floury seeds were first selected from the F_2_ population of *flo7* and the *indica* variety Pei’ai64. More than 170 polymorphic simple sequence repeat markers evenly distributed over the whole genome were selected. To fine map the *flo7* locus, molecular markers were developed based on the nucleotide polymorphisms in the corresponding regions between Nipponbare and cultivar 93-11 (Supplementary Table S1 at *JXB* online).

### Plasmid construction and rice transformation

For complementation of the *flo7* locus, the wild-type *FLO7* cDNA sequence was cloned using primers 5′-GTAGAAGAGG TACCCGGGTTCGCCTCCAGTGCTCGC-3′ and 5′-CTCTAGA GGATCCCCGGGCACAGAGAACACTCCTTCAATACGC-3′, and the PCR product was inserted into the binary vector pCAMBIA2300 under the control of the *ACTIN1* promoter to generate the transformation cassette *pAct1:FLO7.* To generate *pUbi:FLO7-GFP* transgenic plants, the 1092bp cDNA sequence of *FLO7* was inserted downstream of the *UBIQUITIN1* promoter and translationally fused with the green fluorescent protein (GFP) tag at its C terminus in vector pCAMBIA1305.1. The plasmids *pAct1:FLO7* and *pUbi:FLO7-GFP* were introduced into the *flo7* mutant by *Agrobacterium tumefaciens*-mediated transformation as described previously (Jeon *et al.*, 2000). Twenty-eight independent transgenic lines carrying *pAct1:FLO7* and 18 independent transgenic lines carrying *pUbi:FLO7-GFP* were obtained.

### Antibody preparation

For the detection of FLO7 and flo7 proteins in wild type and *flo7* mutant plants, polyclonal antibodies FLO7-C and FLO7-N were produced, respectively. For anti-FLO7-C antibodies, the polypeptide PNDEVNEHHRRD was synthesized and conjugated with keyhole limpet haemocyanin (KLH) to form KLH–PNDEVNEHHRRD. Subsequently, the conjugate was injected into rabbits to produce the polyclonal FLO7-C antibodies (Abmart). For anti-FLO7-N antibodies, a glutathione *S*-transferase-fused polypeptide (aa 1–231 of FLO7) was expressed in *BM*Rosetta (DE3) cells, purified, and injected into rabbits to produce polyclonal FLO7-N antibodies (Abclonal). Anti-HSP82 antibodies were used as a loading control (Beijing Protein Innovation).

### Sequence and phylogenetic analysis

Sequence analysis and homologs of FLO7 were identified using the BLASTP search program of the National Center for Biotechnology Information (http://www.ncbi.nlm.nih.gov). A neighbor-joining tree was constructed using MEGA 5.0 (http://www.megasoftware.net) by the bootstrap method with 1000 replicates (Tamura *et al.*, 2011). The amino acid sequences of FLO7 homologous proteins were aligned using CLUSTALX (http://www.clustal.org).

### RNA isolation and real-time reverse transcription (RT)-PCR

Total RNA was prepared from several plant parts (root, stem, leaf sheath, leaf, panicle, embryo, endosperm, and pericarp) using an RNAprep Pure Plant kit (Tiangen Biotech, Beijing, China). For pericarp, embryo, and endosperm sample preparation, the rice grains were first dehulled using fine-point tweezers. Subsequently, the pericarp was removed using the fine-point tweezers and collected as the pericarp sample. The embryo was obtained by extruding the grain top, while the rest was gathered as the endosperm sample. A 2 μg portion of total RNA was reverse transcribed by priming with oligo(dT_18_) in a 40 μl reaction based on the PrimeScript Reverse Transcriptase kit (TaKaRa, http://www.takara-bio.com). The primers used in this analysis are listed in Supplementary Table S1. The value obtained for *UBQ* (*Os03g0234200*) mRNA was used as an internal control. The real-time RT-PCR primers used to assay starch synthesis-related gene expression have been documented elsewhere (She *et al.*, 2010).

### Subcellular localization of FLO7 protein

To determine the subcellular localization of FLO7, the FLO7 protein sequence was cloned in frame in front of the GFP-coding region in the pAN580-GFP vector to create FLO7–GFP, FLO7^1–69^–GFP, FLO7^Δ1–69^–GFP, and flo7–GFP under the control of the cauliflower mosaic virus (CaMV) 35S promoter. All transient expression constructs were transformed separately into rice protoplasts and incubated in the dark at 28 °C for 16h before examination according to protocols described previously (Chiu *et al.*, 1996; Chen *et al.*, 2006). Fluorescent images were observed using a Zeiss LSM710 confocal laser microscope. *pUbi:FLO7-GFP* was transformed into *flo7* mutant plants. The endosperm from T_1_ transgenic plants was used for observation.

### Protein extraction and western blot analysis

Proteins were extracted in extraction buffer consisting of 50mM Tris/HCl, pH 8.0, 0.25M sucrose, 2mM DTT, 2mM EDTA, and 1mM phenylmethylsulphonyl fluoride, as described previously (Han *et al.*, 2012). Proteins were resolved by SDS-PAGE and transferred electrophoretically to polyvinylidene difluoride membrane (0.45 μm; Millipore, http://www.millipore.com). The membrane was then incubated with antibodies and visualized using ECL Plus reagent (GE Healthcare).

### Histochemical β-glucuronidase (GUS) assays

A putative 1.7kb genomic fragment upstream of the ATG start codon was amplified by PCR using the following primers: 5′-GAATTCCCGGGGATCCGTCCAATACTCTTCAACTG CG-3′ and 5′-GGCCAGTGCCAAGCTTGCGGATATAGCCTAG CCTACTA-3′. The PCR product was cloned into the binary vector pCAMBIA1381. Ten independent transgenic rice lines were produced by *A. tumefaciens*-mediated transformation as described previously (Jeon *et al.*, 2000). Samples for GUS staining were placed in staining solution [50mM Na_3_PO_4_, pH 7.2, 1mM K_3_Fe(CN)_6_, 1mM K_4_Fe(CN)_6_, 1mM 5-bromo-4-chloro-3-indolyl-β-d-glucuronic acid, and 0.1% Triton X-100] and incubated at 37 °C. The stained tissues were photographed using a Canon camera (50D) and enhanced using Photoshop 6.0 (Adobe).

### RNA *in situ* hybridization analysis

A gene-specific region of the coding region of *FLO7* was amplified and cloned into pGEM-T Easy vector (Promega; primers are listed in Supplementary Table S1. *In situ* hybridization was performed according to a previous description (Coen *et al.*, 1990).

### Transient expression analysis in *Nicotiana benthamiana*

The two constructed binary vectors pCAMBIA1305-FLO7-GFP and pCAMBIA1305-flo7-GFP were introduced into the *Agrobacterium* strain EHA105 and used to infiltrate *N. benthamiana* leaves, as described previously (Waadt and Kudla, 2008). *N. benthamiana* protoplasts were isolated as described previously (Ren *et al.*, 2014). Fluorescence images were observed using a Zeiss LSM710 confocal laser microscope. Primers are listed in Supplementary Table S1.

### Luciferase activity assay

The *FLO7* and *flo7* full-length cDNAs were cloned into the vector pGreenII 0800-LUC containing the firefly luciferase gene (*LUC*) to generate *FLO7–LUC* and *flo7–LUC*. Under the control of the CaMV 35S promoter, *FLO7-LUC* and *flo7-LUC* recombinant plasmids were used for transient expression assays. The relative luciferase activity assays were performed as described previously (Zhou *et al.*, 2013a). Primers used in this assay are listed in Supplementary Table S1.

### Yeast two-hybrid assay

*FLO7* was cloned into pGBKT7 (Clontech, http://www.clontech.com), while the genes used in this assay were cloned into pGADT7 (Supplementary Table S1). Yeast transformation and screening procedures were performed according to the manufacturer’s instructions (Clontech).

### Enzyme assays and native PAGE/activity staining

Developing endosperm samples (100mg) harvested at 9 DAF were homogenized on ice in 1ml buffer [50mM HEPES/NaOH, pH 7.4, 2mM MgCl_2_, 12.5% (v/v) glycerol]. The homogenate was centrifuged at 20 000*g* for 10min at 4 °C and the supernatant was used for enzyme activity assays. The enzyme assays and activity staining were performed as described previously (Han *et al.*, 2012; Peng *et al.*, 2014).

## Results

### Phenotypic characterization of the *flo7* mutant

**Fig. 1.**
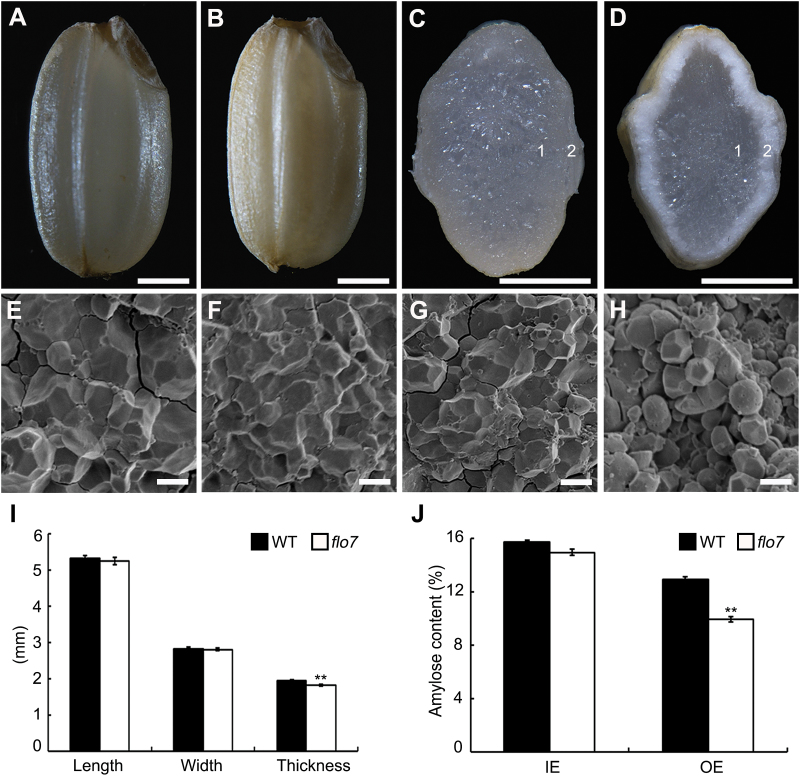
Phenotypic analyses of the *flo7* mutant. (A–D) Appearance and transverse sections of representative wild-type (A, C) and *flo7* mutant (B, D) dry seeds. Bars, 1mm. (E–H) Scanning electron microscopy images of transverse section of the wild-type (E, F) and *flo7* mutant (G, H) grains. (E) and (F) represent the magnified region indicated by ‘1’ and ‘2’ in the wild-type endosperm, respectively, and (G) and (H) represent the magnified region marked by ‘1’ and ‘2’ in the *flo7* mutant endosperm, respectively. Bars, 5 μm. (I) Measurement of seed length, seed width, and seed thickness of wild-type and *flo7* mutant grains (*n*=20 each). (J) Amylose content comparison of the wild-type and *flo7* mutant endosperm parts (*n*=3 each). IE, inner endosperm, OE, outer endosperm. Data are given as means±SD (from at least three independent samples) and were compared with wild type by Student’s *t*-test (**P<0.01).

To identify new regulators of endosperm development, we isolated an endosperm development defective mutant named *flo7* from a mutant pool (in the *japonica* variety Nipponbare background). Throughout the vegetative growth stage, the *flo7* mutant plants displayed no significant differences from the wild-type plants. After fertilization, the *flo7* mutant exhibited a markedly slower grain-filling rate and eventually produced an opaque endosperm, which had an ~13% reduction in grain weight (Fig. 1A, B and Supplementary Fig. S1 at *JXB* online). Notably, cross-section analysis showed that the peripheral region of *flo7* mutant grain appeared floury-white, while the inner endosperm was translucent, as in wild-type endosperm (Fig. 1C, D). Furthermore, scanning electron microscopy analysis revealed that the peripheral endosperm cells of *flo7* mutant were packed with loosely arranged compound starch grains with large spaces, markedly different from the densely packed, irregularly polyhedral starch grains inside the inner endosperm cells of *flo7* mutant as well as those in wild-type cells (Fig. 1E–H). Grain size measurements showed that both seed length and seed width were largely comparable between the wild type and *flo7* mutant, but the seed thickness was significantly reduced in the *flo7* mutant (Fig. 1I). Consistent with the phenotypic characteristics of the *flo7* mutant, the amylose content of the *flo7* mutant in the periphery exhibited a significant reduction compared with the interior endosperm (Fig. 1J). The structural changes in amylopectin were also analyzed. As shown in Supplementary Fig. S2 at *JXB* online, the outer but not the inner parts of the *flo7* endosperm contained a higher proportion of short chains in the range of degree of polymerization (DP) of 8–14 and a lower proportion of both the short chains and the long chains composed of DP 6 and 7 and DP 15–55, respectively, suggesting that amylopectin synthesis was seriously disrupted only in the outer parts of the *flo7* endosperm. These results collectively suggested that the mutation in the *FLO7* gene causes a defect in starch accumulation, which is particularly limited to the periphery of the endosperm during seed development.

### Amyloplast development is affected in the *flo7* endosperm

**Fig. 2.**
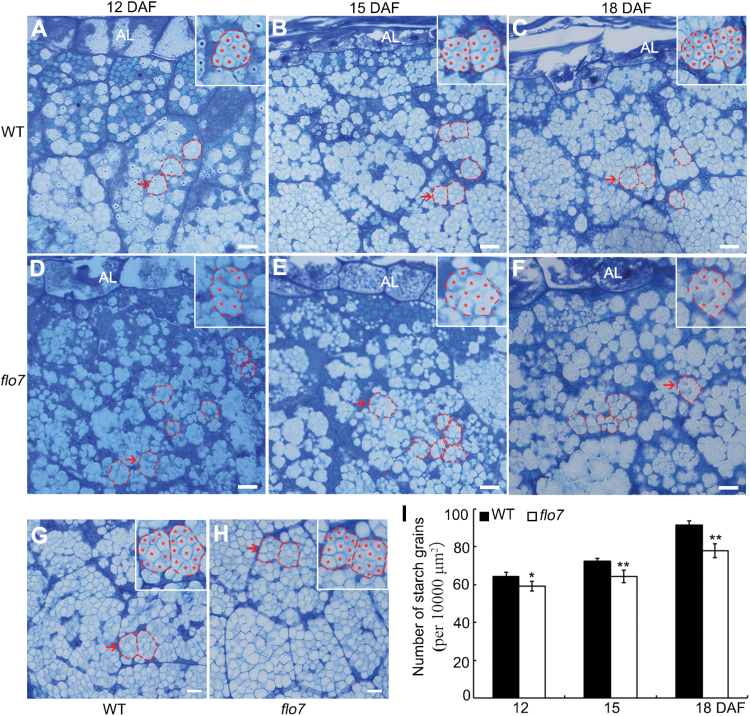
Abnormal compound starch grain development in *flo7* endosperm cells. (A–C) Toluidine blue staining showing the developmental process of wild-type developing endosperm at 12 (A), 15 (B), and 18 (C) DAF, respectively. (D–F) Toluidine blue staining showing the developmental process of *flo7* developing endosperm at 12 (D), 15 (E) and 18 (F) DAF, respectively. The insets represent magnified images of the selected areas in (A) to (F). The amyloplasts are outlined with red dashed lines, while starch granules are indicated by red asterisks. Al, aleurone layer. (G, H) Overview of the developing endosperm interior at 18 DAF in wild type (G) and *flo7* mutant (H) seeds, respectively. The insets represent the magnified images of the selected areas in (G) and (H). (I) Quantitative comparison of amyloplast numbers at various developmental stages in the wild type and *flo7* mutant. Data are given as means±SD (from at least three independent samples) and were compared with wild type by Student’s *t*-test (*P<0.05, **P<0.01). Bars, 10 μm.

To search for the immediate cause of abnormal starch accumulation in the *flo7* mutant, we prepared semi-thin sections of the wild-type and *flo7* mutant developing endosperms at 12, 15, and 18 DAF. In rice endosperm, the cellular compartment for starch synthesis and storage is a type of specially differentiated plastid, termed the amyloplast, where starch is synthesized as small insoluble granules, which, through assembly, swelling and extrusion, form several dozen sharp-edged, polyhedron-filled compound starch grains (Jane *et al.*, 1994; Yun and Kawagoe, 2010). As shown in Fig. 2A–C, toluidine blue staining showed that, in the periphery of wild-type endosperm cells, amyloplasts (outlined by red dashed lines) were filled with densely packed, polyhedral starch granules (faint staining parts, asterisks), indicating that the amyloplasts were well developed. However, in the *flo7* mutant, we found that starch granules (asterisks) within the amyloplasts lacked the clear definition of the wild-type starch granules and appeared more disordered (Fig. 2D–F). By contrast, compound starch grains located in the endosperm interior were largely comparable between the wild type and *flo7* mutant (Fig. 2G, H). In addition, statistical analysis revealed a reduction in the number of amyloplasts in the periphery of the *flo7* mutant endosperm, and such differences became more evident as the endosperm developed (Fig. 2I).

**Fig. 3.**
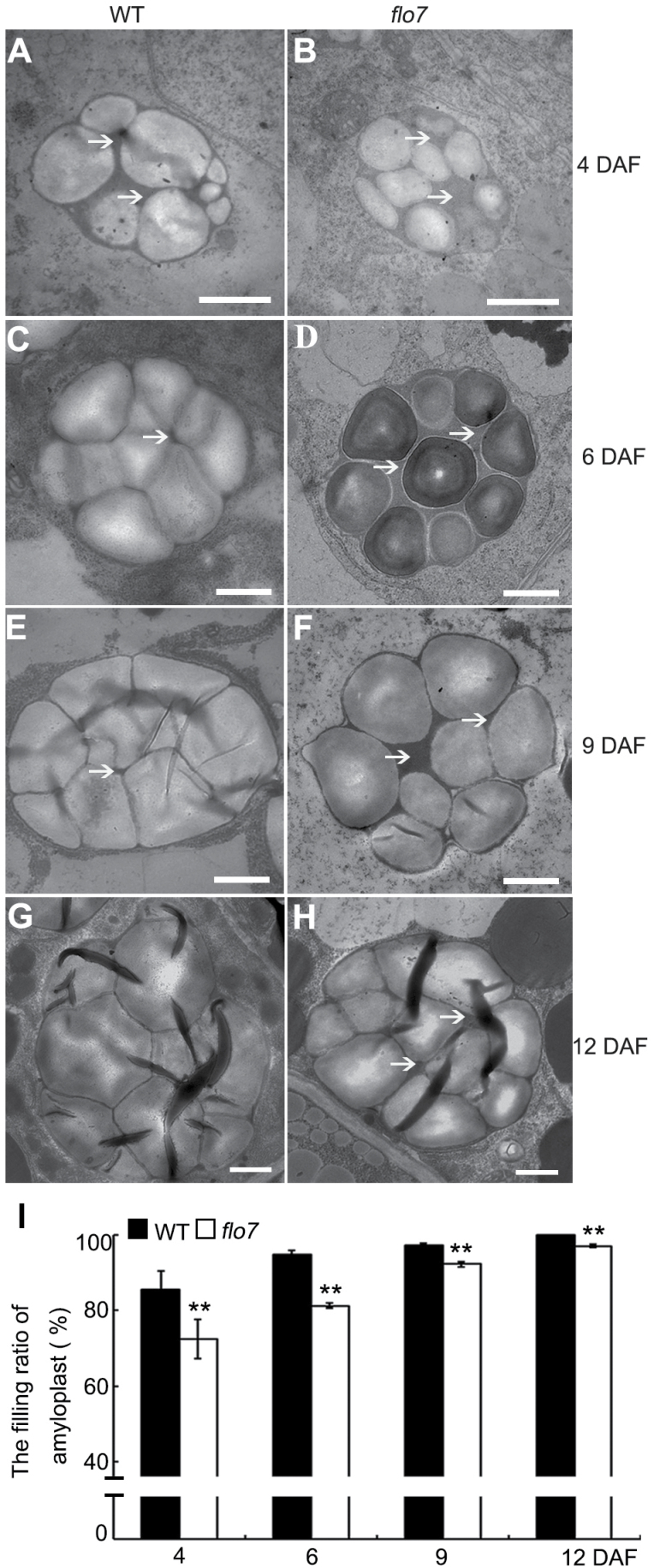
Electron micrographs depicting amyloplast development in the developing endosperm periphery of the wild type and *flo7* mutant. (A–H) Transmission electron microscope analysis of the compound starch grains of the wild type and *flo7* mutant at 4 (A, B), 6 (C, D), 9 (E, F), and 12 (G, H) DAF. White arrows indicate the stroma inside the amyloplast. Bars, 1 μm. (I) Filling ratio of amyloplasts in developing wild-type and *flo7* mutant endosperm at 4, 6, 9, and 12 DAF (*n*=40 each). Data are given as means±SD (from at least three independent samples) and were compared with wild type by Student’s *t*-test (**P<0.01).

To better dissect the developmental defect in the compound starch grains in the *flo7* mutant, we performed transmission electron microscopy analysis with ultrathin sections prepared from chemically fixed peripheral endosperm cells at earlier developmental stages (4, 6, 9, and 12 DAF). As observed in the wild type, small round starch granules were initially formed in the stroma of the amyloplasts, and quickly occupied the amyloplast interior, eventually forming the sharp-edged polyhedron (Fig. 3A, C, E, G). In contrast to the densely arranged starch granules in wild type at 6 DAF and thereafter, the stroma surrounding starch granules in the *flo7* mutant at the same developmental stages were evidently larger (Fig. 3B, D, F, H), implying that a growth arrest of amyloplasts occurred in the *flo7* mutant. Such a difference between wild type and *flo7* mutant was further evaluated by the statistical analysis of the filling ratio of the amyloplasts (Fig. 3I). Together, these results suggested that the *FLO7* gene may play a vital role in compound starch grain development in the endosperm periphery during the grain-filling stage.

### Map-based cloning and complementation of the *FLO7* gene

**Fig. 4.**
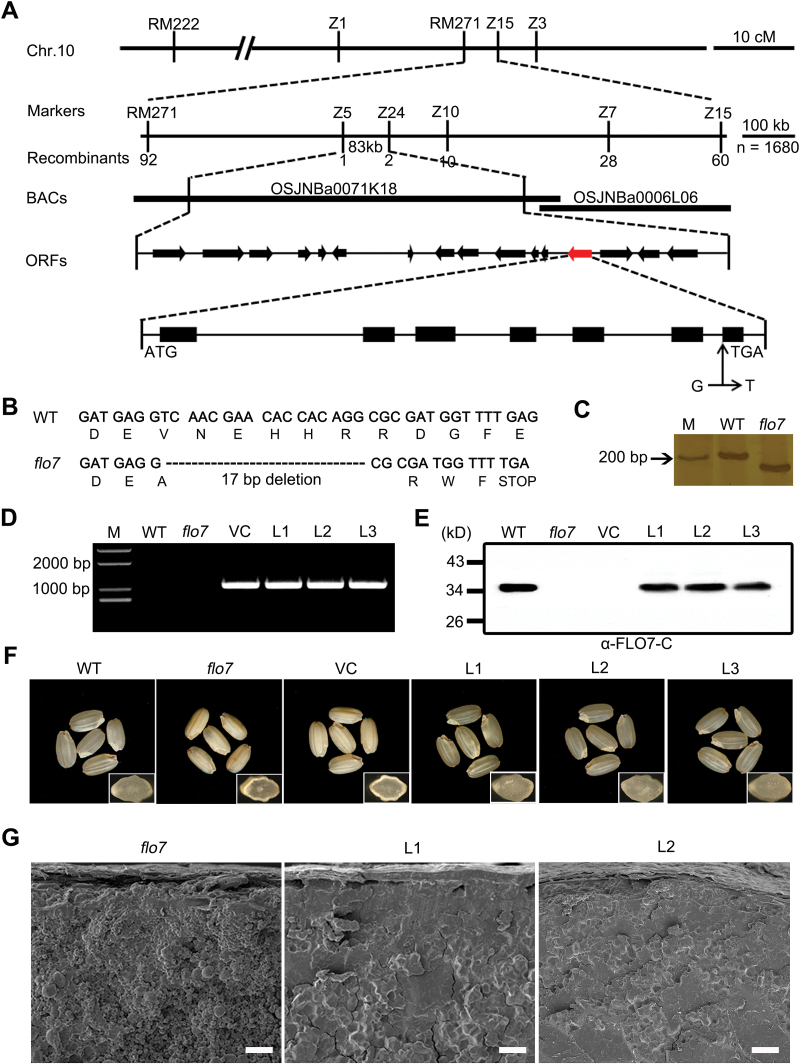
Map-based cloning and complementation of the *flo7* mutant. (A) Fine mapping of the *FLO7* locus (red arrow). The molecular markers and number of recombinants are shown. (B) cDNA sequence comparison showing the 17bp deletion in the *flo7* mutant. (C) Identification of the 17bp deletion between the wild type and *flo7* mutant using an InDel marker. M, marker. (D) Molecular identification of transgenic plants by PCR amplification. Primer pairs in the vector were used to test the positive transgenic lines. M, marker; VC, vector control. (E) Western blot analysis showing that full-length FLO7 protein is present in the developing seeds of wild-type and complemented lines but is absent in the *flo7* mutant and vector control. (F, G) Functional complementation of the *FLO7* gene completely rescues the grain appearance (F) and compound starch grain arrangement (G) of the *flo7* mutant. Insets in (F) represent the transverse sections of representative grains. Bars, 20 μm.

The *flo7* mutant was initially isolated from a screening of the T-DNA inserted mutant pool in *japonica* variety Nipponbare. Unfortunately, the phenotypic defect was not linked to the T-DNA insertion site. To clarify the molecular mechanism controlling the *flo7* phenotypes, a map-based cloning approach was used to isolate the *FLO7* gene. We first crossed the *flo7* mutant with the *indica* variety Pei’ai64 to generate an F_2_ mapping population. The *flo7* mutation locus was mapped in a 4.1 cM interval between simple sequence repeat marker RM271 and insertion/deletion (InDel) marker Z15 on the long arm of chromosome 10, based on 10 recessive *flo7* individuals. Furthermore, by using a total of 1680 recessive individuals, the *flo7* locus was narrowed down to an 83kb region between InDel markers Z5 and Z24 on the BAC clone OSJNBa0071K18 (Fig. 4A), which included 16 putative ORFs (http://rice.plantbiology.msu.edu/). Comparison with the wild-type genomic sequences revealed that *Os10g0463800* in the *flo7* mutant contained a single nucleotide substitution of guanine (G) to thymidine (T), which was located at the splicing acceptor site of the 6th intron. cDNA sequencing further showed that this replacement resulted in a 17bp splicing deletion and thus produced an aberrant transcript. If translated, this transcript would encode a protein, named flo7, which is missing the last 32 aa of Os10g0463800, but has an additional 4 aa resulting from the frame-shift mutation (Fig. 4B, C).

To test whether *Os10g0463800* corresponded to the candidate *FLO7* gene, a nucleotide fragment containing the *FLO7* coding sequence driven by an *ACTIN1* promoter was introduced into the *flo7* mutant. Positive transgenic lines expressing *pAct1:FLO7* were identified by PCR and western blot analysis (Fig. 4D, E). Three independent transgenic lines showed the complete rescue of the endosperm developmental defects, including grain appearance and compound starch grain arrangement (Fig. 4F, G). In summary, we concluded that *Os10g0463800* is the gene responsible for the *flo7* phenotype.

### *FLO7* encodes a green-plant-unique protein belonging to the DUF1338 superfamily

The *FLO7* gene was predicted to encode a protein composed of 364 aa that harbors a putative plastid-targeting signal in its N-terminal region and a DUF1338 domain in the middle and C terminus (aa 70–355; http://smart.embl-heidelberg.de) (Supplementary Fig. S3A at *JXB* online). Apart from these, no other functional domains were identified in the FLO7 protein. To understand further the phylogenetic relationship of FLO7-related proteins in plants and other eukaryotes, we searched and compared the predicted protein sequences from 13 phylogenetically different organisms. Based on the phylogenetic analysis, *FLO7* seems to be a green-plant-unique gene and its orthologous genes widely exist as single copies in green algae, bryophytes, pteridophytes, and higher plants (Supplementary Fig. S3B), further supporting the possibility that rice *FLO7* is the evolutionary product of green algae *FLO7*. However, so far none of these genes have been functionally characterized, including the *FLO7* homolog in the model plant *Arabidopsis*. Notably, the mutation in the *flo7* mutant partially removed the DUF1338 domain and thus led to an incomplete DUF1338 domain lacking the C-terminal 23 aa, which are highly conserved among green plants (Supplementary Fig. S3C). Given the phenotypic defect of the *flo7* mutant, it seems that these missing 23 aa are critical for FLO7 protein function.

### Expression pattern of the *FLO7* gene

**Fig. 5.**
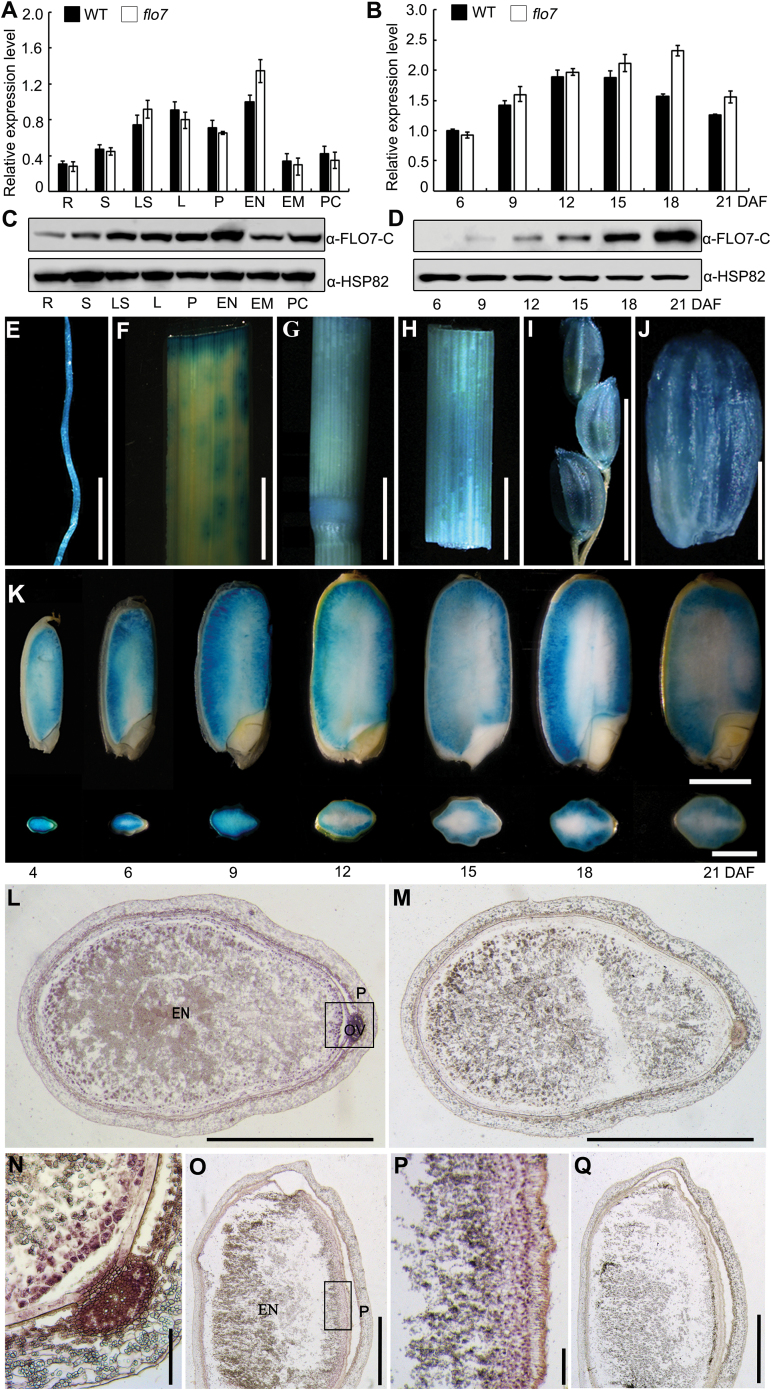
Expression pattern of *FLO7.* (A, B) Expression levels of *FLO7* in the various tissues and in the developing endosperms of the wild type and *flo7* mutant. *UBQ* was used as an internal control. Values are means±SD. R, root; S, stem; LS, leaf sheath; L leaf; P, panicle; EN, endosperm; EM, embryo; PC, pericarp and seed coat. (C, D) Western blot analysis showing that FLO7 protein is present in various tissues tested and in the developing endosperms. HSP82 antibodies were used as a loading control. (E–J) GUS activity in root (E), leaf (F), nodes (G), leaf sheaths (H), panicle (I), and the grain at 9 DAF (J). Bars, 1cm (E–I); 2mm (J). (K) GUS activity at the different filling stages of the endosperm (4–21 DAF). Vertical sections of the grains (upper images) and a cross-section of the grains (lower images) are shown. Bars, 2mm. (L–Q) RNA *in situ* hybridization in the endosperm. (L, M) Transverse section of the endosperm at 12 DAF. (N) Enlarged view of the rectangular region in (L). (O, Q) Longitudinal section of endosperm at 12 DAF. (P) Enlarged view of the rectangular region in (O). In (M) and (Q), the sense probe was used for hybridization as a negative control. EN, endosperm, P, pericarp, OV, ovular vascular trace. Bars,1mm (L, M, O, Q); 100 μm (N, P).

To test whether *FLO7* is expressed specifically in the endosperm or is also broadly expressed in other organs, we analyzed the *FLO7* expression pattern. We first performed real-time RT-PCR analysis and found that *FLO7* was expressed constitutively in all tested organs, including root, stem, leaf sheath, leaf, panicle, and endosperm, with the lowest and the strongest level of expression being detected in the root and endosperm, respectively (Fig. 5A). Next, we examined the expression profile of *FLO7* in more detail at various developmental stages of endosperms. We found that the expression of *FLO7* peaked at approximately 12 DAF and then gradually declined from 15 DAF (Fig. 5B). Notably, we observed an apparent increase in expression of the aberrant *flo7* transcript in comparison with the wild-type *FLO7* gene across endosperm development (Fig. 5B), most likely due to a positive-feedback regulation. To better characterize the tissue distribution profile of FLO7 proteins, antibodies were raised against a peptide located in the C-terminal region of FLO7 (anti-FLO7-C; Supplementary Fig. S4 at *JXB* online). As shown in Fig. 4E, the FLO7 antibodies specially recognized the putative 35kDa endogenous FLO7 protein band in wild type but not in the *flo7* mutant. Western blot analysis of protein extracts from various tissues demonstrated that FLO7 was expressed in all of the tissues examined (Fig. 5C). During grain development, FLO7 protein was first detectable at 9 DAF and the level continuously increased as the grain developed (Fig. 5D). The inconsistency between RNA level and protein accumulation suggests that FLO7 proteins may be extremely stable and resistant to protease degradation, implying its essential role in the late stage of endosperm development in rice.

Strikingly, a *FLO7* promoter-driven GUS reporter gene assay revealed that *FLO7* was expressed at a relatively high level in the periphery of the endosperm, while only a very weak signal was detected in the central region of the endosperm (Fig. 5E–K). Given that the predicted promoter used for the fusion studies might miss some important *cis*-elements, RNA *in situ* hybridization assay was performed with the developing endosperm at 12 DAF, which revealed that the peripheral cell layers of the endosperm displayed strong labeling whereas no specific signals were detected in the central region (Fig. 5L–Q), further confirming the essential role for *FLO7* in the development of the endosperm periphery. In summary, we concluded that *FLO7* is a constitutively expressed gene with specific expression in the periphery of the endosperm.

### FLO7 localizes to the stroma of amyloplasts

**Fig. 6.**
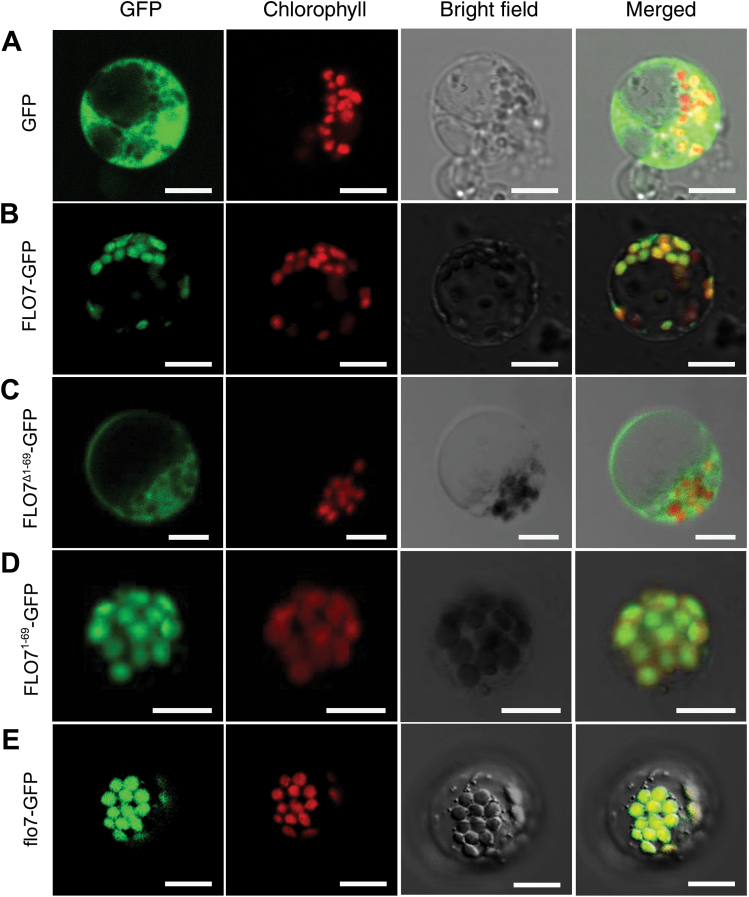
) Confocal microscopy images showing the subcellular localization of FLO7 in rice protoplasts. (A) GFP by itself localized to the cytoplasm and nucleus. (B) Full-length FLO7 fusion protein (FLO7–GFP, aa 1–364) localized to the chloroplasts. (C) FLO7 fusion protein lacking the N-terminal 69 aa (FLO7^Δ1–69^–GFP; aa 70–364) displayed a diffuse localization pattern. (D) The N-terminal 69 aa of FLO7 (FLO7^1–69^–GFP) were capable to target GFP proteins to the chloroplasts. (E) Mutation of the FLO7 protein in the *flo7* mutant (flo7–GFP, aa 1–336) failed to impair its chloroplast-localization pattern. After 16h of incubation, rice protoplasts were observed using a confocal laser scanning microscope. GFP (green), chlorophyll autofluorescence (red), bright-field images, and an overlay of green and red signals are shown. Bars, 10 μm.

ChloroP (Emanuelsson *et al.*, 2007) and WoLF PSORT (Nakai and Horton, 2007) prediction analyses revealed that the FLO7 protein contains a chloroplast-targeting transit peptide in the N terminus. To verify the chloroplast-localized pattern and characterize the chloroplast-targeting signal of FLO7, four GFP fusions of the wild-type and truncated FLO7 proteins, FLO7–GFP, FLO7^1–69^–GFP, FLO7^Δ1–69^–GFP, and flo7–GFP, were constructed and transiently expressed in rice protoplasts. As expected, GFP alone was distributed evenly in the cytoplasm and nuclei, whereas the wild-type FLO7–GFP fusion proteins co-localized exclusively with the autofluorescent signals of chlorophyll in the chloroplasts characterized by a small disc pattern (Fig. 6A, B). Furthermore, confocal microscopy observation showed that, compared with the FLO7^Δ1–69^–GFP fusion protein lacking the N terminus, which had a cytosol-localized pattern, FLO7^1–69^–GFP was efficiently targeted to the chloroplasts, suggesting that the N-terminal 69 aa are essential for its targeting to the chloroplast (Fig. 6C, D). Supporting this hypothesis, the flo7 mutant protein lacking the C-terminal 32 aa remained in the chloroplast (Fig. 6E).

**Fig. 7.**
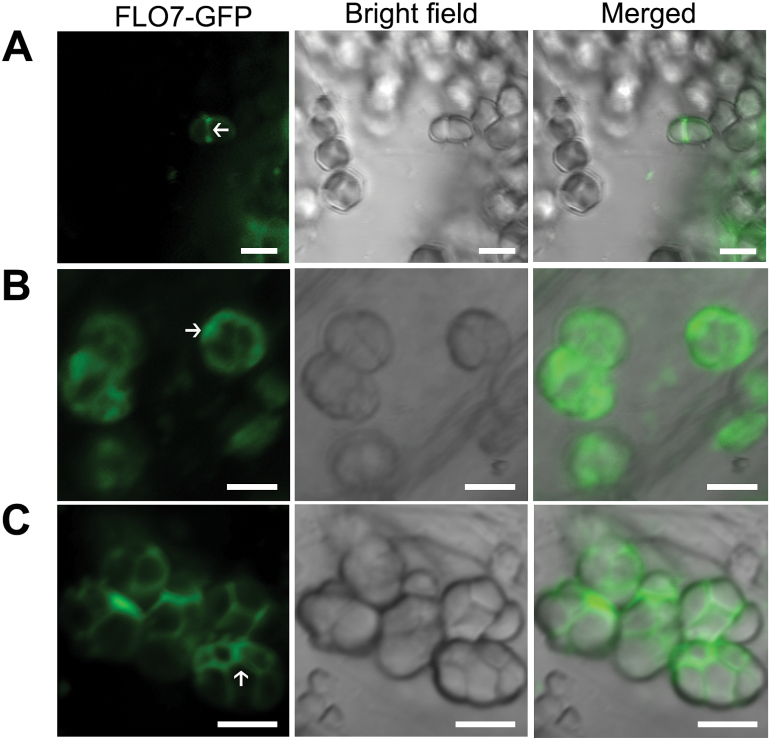
Amyloplast localization of FLO7–GFP in endosperm cells. Confocal microscopy images showing the localization of FLO7–GFP in developing endosperm at 2 (A), 4 (B) and 6 (C) DAF. The stroma in the amyloplast is indicated by white arrows. Bars, 5 μm.

To verify the subcellular distribution pattern of FLO7 *in vivo*, we generated transgenic rice lines expressing *FLO7–GFP* under the control of maize *UBIQUITIN1* promoter (*pUbi*), in a *flo7* mutant background, and found that the grain developmental defects of the *flo7* mutant were restored to the wild-type appearance (Supplementary Fig. S5A, B at *JXB* online), demonstrating that FLO7–GFP was functional. Fluorescence microscopy revealed that FLO7–GFP proteins were localized to the chloroplasts and amyloplasts in leaf sheath cells and in developing endosperm cells, respectively (Supplementary Fig. S5C, D). To further determine the detailed distribution of FLO7–GFP inside amyloplasts, we observed the developmental process of amyloplasts expressing FLO7–GFP fusions. As shown in Fig. 7, FLO7–GFP fluorescent signals accumulated mainly in the inter-starch granule space (corresponding to the stroma), which was similar to the previously reported stroma-targeted tpCherry rather than the amyloplast membrane-localized BT1–GFP (Yun and Kawagoe, 2010). Together, these data suggested that FLO7 is a stroma-localized protein with an N-terminal plastid-targeting signal.

### The C-terminal 32 aa are essential for stable accumulation of FLO7 protein

**Fig. 8.**
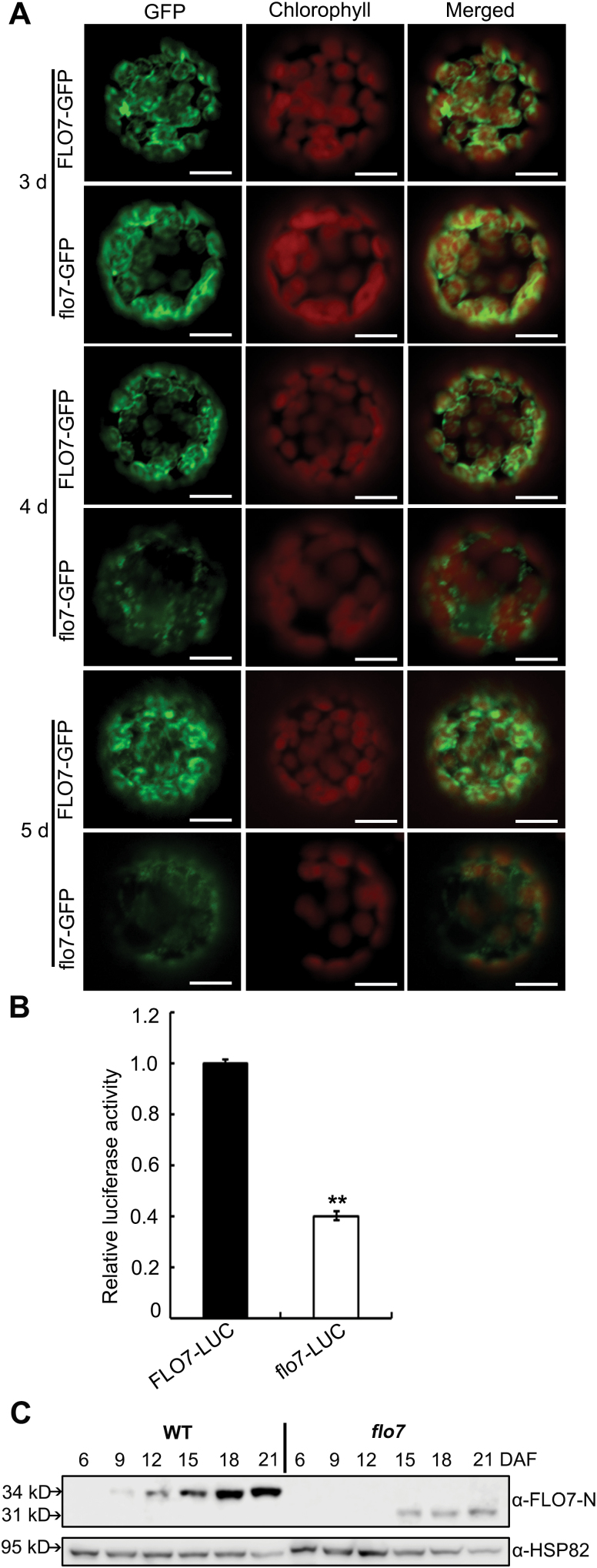
Stability test of the flo7 protein. (A) Confocal microscopy images showing the degradation pattern of flo7–GFP fusion protein in *N. benthamiana* leaf-derived protoplasts. Bars, 10 μm. (B) Relative luciferase activity of FLO7–LUC and flo7–LUC in rice protoplasts. Data are given as means±SD of three independent experiments. All data were compared with FLO–LUC by Student’s *t*-test (**P<0.01). (C) Western blot analysis of FLO7 accumulation in the developing endosperm of the wild type and *flo7* mutant. Anti-HSP82 antibodies were used as a loading control.

To test whether the mutation in *flo7* affected the stability of the FLO7 protein, we first transiently separately expressed *FLO7–GFP* and *flo7–GFP* driven by the CaMV 35S promoter in *N. benthamiana* leaves, followed by protoplast isolation 3 d after infiltration. Fluorescence microscopy observation confirmed that both FLO7–GFP and flo7–GFP exhibited the same chloroplast-localized pattern in *N. benthamiana* protoplasts as that displayed in rice protoplasts (Fig. 8A). Interestingly, we noted that, 4 d after infiltration and thereafter, the fluorescence signals from the flo7–GFP fusion protein were weaker compared with those from FLO7–GFP at the same time points (Fig. 8A). Furthermore, a luciferase activity assay, performed in a rice protoplast system, showed that the accumulation of flo7–LUC fusion proteins was significantly lower than wild-type FLO7–LUC (Fig. 8B). As the above-described antibodies against FLO7 failed to recognize the truncated flo7 mutant protein (Fig. 4E), another antibody was raised against the N-terminal region of FLO7 (anti-FLO7-N; Supplementary Fig. S4 at *JXB* online). As shown in Fig. 8C, the anti-FLO7-N antibodies specially recognized the wild-type FLO7 protein band and revealed a similar accumulation pattern in developing endosperm to the C-terminal region-detected antibodies (Fig. 5D). Notably, the N terminus-recognizing antibodies were able to detect the putative 31kDa flo7 protein, although the accumulation level of *flo7* mutant proteins appeared obviously lower than in wild type (Fig. 8C). Together, these results suggested that lack of the C-terminal 32 aa significantly interfered with the stable accumulation of flo7 protein.

## Discussion

### *flo7* is a novel endosperm-defective mutant with distinct characteristics

Rice endosperm has been an excellent system to elucidate how gene networks regulate starch synthesis and amyloplast development, by screening for opaque-kernel mutants (Nelson and Pan, 1995; Satoh and Omura, 1981). Waxy or dull mutants produce opaque endosperm after dehydration due to air spaces in the inter- or intra-starch grains, while other mutants exhibit this appearance earlier, most likely resulting from irregularly and loosely packed starch grains. Previous studies have isolated and functionally characterized at least 23 endosperm-defective mutants, including three white-core endosperm mutants (*flo4*, *flo5*, and *rsr1*; Kang *et al.*, 2005; Ryoo *et al.*, 2007; Fu and Xue, 2010), 11 floury endosperm mutants (*flo1* to *flo3*, *ae*, *pdi1-1*, *gpa1* to *gpa3*, *osagpl2-3*, *flo6*, and *ssg4*; Satoh and Omura, 1981; Nishio and Iida, 1993; Matsushima *et al.*, 2010; She *et al.*, 2010; Wang *et al.*, 2010; Han *et al.*, 2012; Zhang *et al.*, 2012a; Liu *et al.*, 2013; Peng *et al.*, 2014; Ren *et al.*, 2014), four shrunken endosperm mutants (*isa1*/*sugary1*, *osagpl2*, *osagps2*, and *pho1*; Kawagoe *et al.*, 2005; Lee *et al.*, 2007; Satoh *et al.*, 2008), three dull/waxy endosperm mutants (*dull1*, *dull3*, and *waxy*; Zeng *et al.*, 2007; Isshiki *et al.*, 2008; Zhang *et al.*, 2012b), and two white belly endosperm mutants (*gif1* and *bzip58*; Wang *et al.*, 2008; Wang *et al.*, 2013). Additionally, the quantitative trait locus *Chalk5* has also been reported to confer grain chalkiness (Li *et al.*, 2014).

In this study, we have reported the isolation and characterization of the *flo7* mutant, which exhibited several distinct features. First, almost all types of previously reported endosperm mutants, such as white-core, floury, dull, and shrunken endosperms, display readily observed phenotypic defects in the endosperm core. As grain filling begins in the core and then spreads to the outer region during endosperm development in rice, it can be speculated that these genes are essential for the early stage of endosperm development in rice. Notably, our *flo7* mutant caused a converse phenotypic variation with a normally developed core but an aberrant periphery (Fig. 1A–H), suggesting the essential role of FLO7 in the late stage of endosperm development in rice. Secondly, despite the fact that most of these defective endosperm mutants display a floury-white appearance, they differ from each other in amyloplast morphology due to damage in different developmental processes of the amyloplast. For example, loss-of-function of *SSG5* seriously inhibits the initiation of starch granules, thus producing amyloplasts lacking an internal compound structure (Matsushima *et al.*, 2010). Mutations in *SSG1/SSG2/SSG3/BEIIb* and *FLO6* develop smaller abnormal amyloplasts, while *ssg4* endosperm contains enlarged amyloplasts (Matsushima *et al.*, 2010; Peng *et al.*, 2014). In contrast, statistical analysis revealed that the size of the amyloplasts was largely comparable in the peripheral endosperm cells of the wild type and *flo7* mutant, but the stroma space in the *flo7* mutant was obviously larger than that in the wild type (Fig. 3A–I), implying that mutation of *FLO7* may restrain the enlargement of starch granules. Thirdly, although our *flo7* mutant also exhibited a lower amylose content, as did the waxy/low-amylose mutants (Fig. 1J), the mechanisms causing the whitish appearance are thought to be different, as waxy and low-amylose mutants become whitish in appearance only after the water content of the grains becomes low, while our *flo7* mutant exhibited a whitish grain appearance earlier, before drying. In addition, the marked peripheral defects of the *flo7* endosperm were most reminiscent of our previously reported *T3612*/*pdi1-1* mutant, which represents a type of rice 57H mutant with defects in endoplasmic reticulum export of storage proteins (Han *et al.*, 2012). However, we failed to detect storage protein sorting defects in the *flo7* mutant. Together, these data suggest that FLO7 defines a novel regulator of starch synthesis and amyloplast development in rice endosperm.

### Expression pattern of the *FLO7* gene

Endosperm development is a complicated and sophisticated process involving starch synthesis and storage protein accumulation. Promoters, as a pivotal part of gene structures, play essential roles in regulating endosperm development temporally and spatially. Previous studies using stable transgenic rice plants have generated a whole body of evidence demonstrating that storage protein accumulation within the endosperm is regulated spatially. For example, in synthesis of glutelin (a predominant component of storage proteins, including three major subfamilies: *GluA*, *GluB*, and *GluC*), the expression of the *GluA1*, *GluA2*, and *GluA3* genes is confined mainly to the peripheral portion of the endosperm, while *GluB5* and *GluC* show an almost evenly distributed pattern within the whole endosperm (Qu *et al.*, 2008). However, the question of whether starch synthesis within the endosperm also exhibits such a spatial accumulation pattern remains to be answered.

In this work, our real-time RT-PCR analysis showed the relatively stable expression of the *FLO7* gene across early to late endosperm development (Fig. 5B), whereas our western blot assays revealed that the FLO7 protein exhibited a continuous accumulation pattern from 9 DAF (Fig. 5D). A possible explanation for this inconsistency between RNA transcription and protein accumulation is that programmed cell death may contribute to the slightly reduced expression of the *FLO7* gene across mid- to late endosperm development, due to disruption of the transcript system. On the other hand, the FLO7 proteins may be highly stable and resistant to protease degradation, and thus are stored to support late endosperm development (i.e. the stage for peripheral endosperm development) and seed germination (see below). These data support the notion that the *FLO7* gene functions mainly in the late stage of endosperm development. Furthermore, the GUS activity assay showed that the *FLO7* promoter can direct GUS expression, especially in the peripheral region of the developing endosperm (Fig. 5K). We then performed RNA *in situ* hybridization and found that the *FLO7* gene indeed exhibited strong and specific expression in the endosperm periphery (Fig. 5L–Q). Previous studies have identified a few genes as regulators required for endosperm core development, such as *FLO2* and *FLO6* (She *et al.*, 2010; Peng *et al.*, 2014). In this study, we identified *FLO7* as a regulator responsible for starch synthesis within the peripheral endosperm, collectively supporting the notion that starch synthesis in rice endosperm is also regulated spatially.

The ovular vascular trace is considered to function in transporting nutrition into the endosperm tissue. It has also been reported that the ovular vascular trace-localized GIF1 protein, a cell-wall invertase, plays a pivotal role in carbon partitioning during the early grain-filling stage. The *gif1* mutant grain displays a slow grain-filling rate and obvious chalkiness, while overexpression of *GIF1* driven by its native promoter produces larger grains (Wang *et al.*, 2008). Interestingly, the hybridization signal of *FLO7* was also readily detected in the ovular vascular trace (Fig. 5L, N), indicating a possible role for *FLO7* in transporting nutrition into endosperm cells. Expression analyses of the *FLO7* transcript and FLO7 protein showed that they exhibited a relatively higher expression level in the leaf (Fig. 5A, C). Furthermore, fluorescence microscopy observation revealed that FLO7–GFP was efficiently targeted to the chloroplasts in the transgenic leaf sheath cells (Supplementary Fig. S5C). These results raise the question of what role FLO7 plays in leaf chloroplast development. Unlike *ssg4* with an easily observed leaf color phenotype, the leaves of the *flo7* mutant showed no obvious difference from the wild-type ones (See Supplementary Fig. S6A, B at *JXB* online). Since ultrastructure observation of amyloplasts in peripheral endosperm cells revealed that starch granule development may be restrained in *flo7* peripheral endosperm cells (Fig. 3), we speculated that the *flo7* mutation may also affect the starch granule development in chloroplasts. As expected, the starch granules but not grana stacks and envelope membranes were seriously affected in the *flo7* mutant compared with those in the wild-type chloroplasts (Supplementary Fig. S6C-G). These data collectively suggest that FLO7 is essential for starch granule development within chloroplasts but is not necessary for chloroplast development and seedling growth. It is also remarkable that, although the combined real-time RT-PCR, western blot, and GUS staining assays showed the broader expression of the *FLO7* gene in tissues other than the endosperm and leaf, we failed to observe obviously phenotypic defects except for slightly delayed germination. One hypothesis is that other genes with a similar function to *FLO7* might offset the loss caused by mutation in the *FLO7* gene in the *flo7* mutant. Together, these data support an essential role of *FLO7* in peripheral endosperm development, and provide insights into the spatial regulation of endosperm development in rice.

### Possible functions of the FLO7 protein

Based on the BLAST search result and phylogenetic analysis, *FLO7* represents a green-plant-unique gene and broadly exists as a single-copy gene (Supplementary Fig. S3B), suggesting its essential role in regulating green plant development. However, *FLO7* homologs of other green plants have not been functionally identified. Consistent with the amyloplast developmental defects in the *flo7* mutant, the functional FLO7–GFP protein localized to the stroma inter-starch granules (Fig. 7). These data suggested that FLO7 plays essential roles in regulating amyloplast development.

Previous studies have shown that mutations of some regulators indirectly involved in starch synthesis usually lead to significant alternations of starch biosynthesis-related gene expression in genes such as *FLO2*, *GIF1*, *RSR1*, and *OsbZIP58* (Wang *et al.*, 2008; Fu and Xue, 2010; She *et al.*, 2010; Wang *et al.*, 2013). To ascertain whether the disrupted gene expression or activity of starch synthesis enzymes contributed to the *flo7* mutant phenotype, starch synthesis-associated gene expression and zymogram analyses were performed using the developing endosperm at 9 DAF (Supplementary Fig. S7 at *JXB* online). The results indicated that neither gene expression nor the activity of starch synthesis enzymes in the *flo7* mutant showed a significant difference from the wild type, suggesting that *FLO7* does not function directly in regulating starch synthesis gene expression or activity. To further dissect the molecular mechanism of FLO7 in starch synthesis, we tested possible protein–protein interactions between FLO7 and starch synthesis enzymes and a few recently reported regulators including FLO2, FLO4, and FLO6, using the yeast two-hybrid approach (Supplementary Fig. S8 at *JXB* online; Kang *et al.*, 2005; She *et al.*, 2010; Peng *et al.*, 2014). Unfortunately, we failed to detect any interaction between FLO7 and those proteins, suggesting that FLO7 may function in starch synthesis in a manner distinct from previously reported proteins. Future studies on searching for FLO7-interacting partners may help us to reveal more details about the regulation of starch synthesis and amyloplasts in green plants.

## Supplementary data

Supplementary data are available at *JXB* online.

**Supplementary Fig. S1.** Time course analysis of the wild-type and *flo7* grain development.

**Supplementary Fig. S2.** Characterization of amylopectin chain length distribution difference between the wild type and *flo7* mutant.

**Supplementary Fig. S3.** Structure and phylogenetic analyses of the FLO7 protein.

**Supplementary Fig. S4.** Polypeptide sequences for FLO7 polyclonal antibody production.

**Supplementary Fig. S5.** Complementation of the *flo7* mutant phenotypes by *UBIQUITIN1* promoter-driven FLO7–GFP.

**Supplementary Fig. S6.** Ultrastructure of chloroplasts in mesophyll cells of 2-week-old wild-type and *flo7* seedlings.

**Supplementary Fig. S7.** Expression levels of the genes involved in production of storage starch as well as zymogram analysis of starch synthesizing enzymes.

**Supplementary Fig. S8.** Yeast two-hybrid assays between FLO7 and proteins involved in starch synthesis.

**Supplementary Table S1.** Primers used in this study.

Supplementary Data
