# Supplementary material for: FLOURY ENDOSPERM7 encodes a regulator of starch synthesis and amyloplast development essential for peripheral endosperm development in rice
Source: J Exp Bot. 2015 Nov 24;67(3):633–47. doi: 10.1093/jxb/erv469 (PMC4737065; doi:10.1093/jxb/erv469)
Supplement: Supplementary Data [file supp_67_3_633__index.html]

 FLOURY ENDOSPERM7 encodes a regulator of starch synthesis and amyloplast development essential for peripheral endosperm development in rice — FLOURY ENDOSPERM7 encodes a regulator of starch synthesis and amyloplast development essential for peripheral endosperm development in rice — Supplementary Data 

# *FLOURY ENDOSPERM7* encodes a regulator of starch synthesis and amyloplast development essential for peripheral endosperm development in rice

## Supplementary Data

Data files

- Supplementary Data - Supplementary Data
