# Supplementary material for: FLOURY ENDOSPERM7 encodes a regulator of starch synthesis and amyloplast development essential for peripheral endosperm development in rice
Source: J Exp Bot. 2015 Nov 24;67(3):633–47. doi: 10.1093/jxb/erv469 (PMC4737065; doi:10.1093/jxb/erv469)
Supplement: Supplementary Data [file supp_erv469_Supplementary_material.pdf]

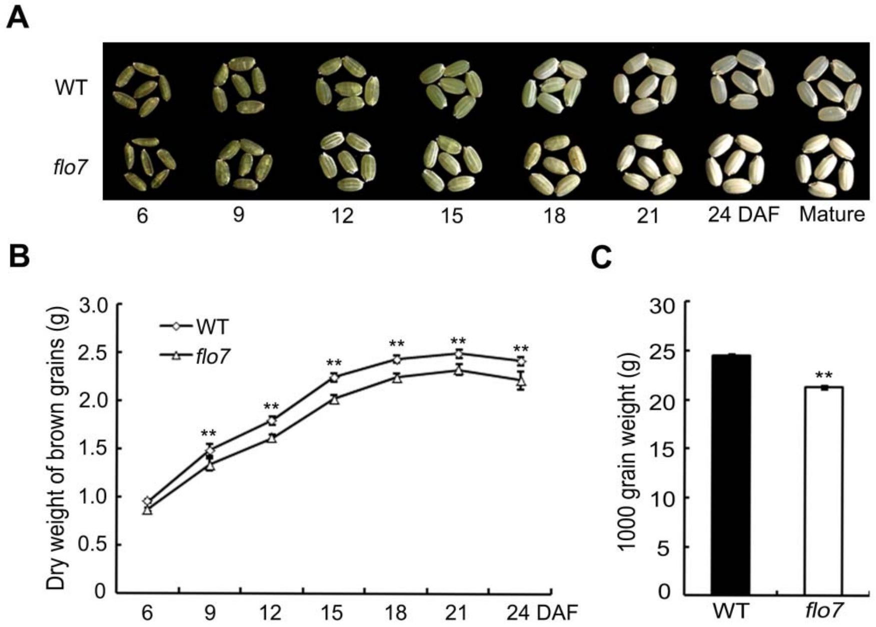

Supplemental Figure S1. Time course analysis of the wild-type and *flo7* grain development.

(A) Grain appearance of the wild-type and *flo7* mutant at series of growth stages as indicated. (B) Grain-filling process of the wild type and *flo7* mutant. Weight indicates the weight of 100 brown grains (n = 3 each). (C) 1000-grain weight of wild type and *flo7* mutant (n = 3 each). DAF, days after fertilization. Data are given as means ± SD and were compared with wild type by Student's t test (**P < 0.01).

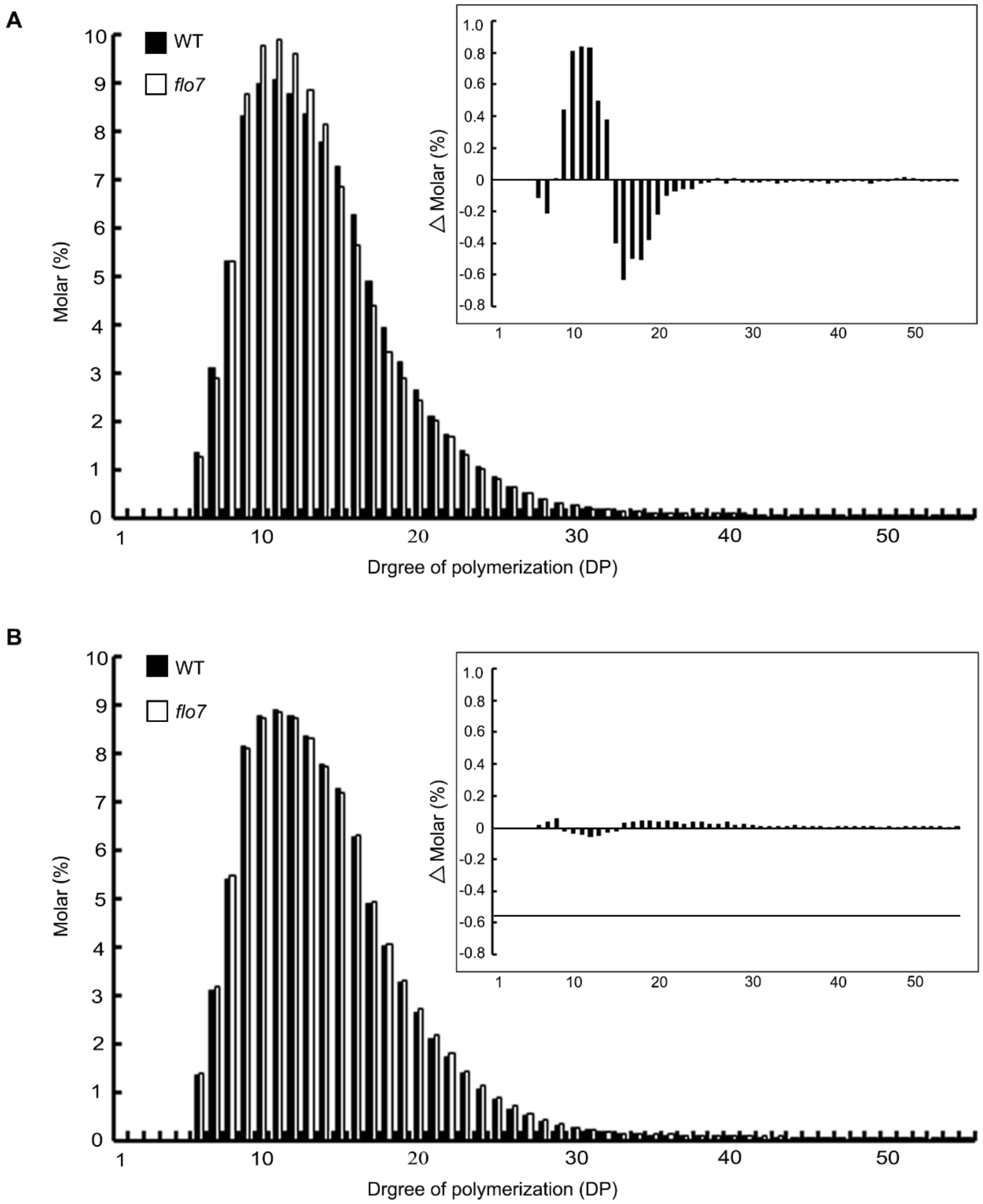

Supplemental Figure S2. Characterization of amylopectin chain length distribution difference between wild type and *flo7* mutant.

(A) Chain length distributions of amylopectin in the outer parts of endosperm from wild type and *flo7* mutant. (B) Chain length distributions of amylopectin in the inner parts of endosperm from wild type and *flo7* mutant. The inset graphs compare the chain length distribution patterns (Δ molar %) between wild type and *flo7* mutant.

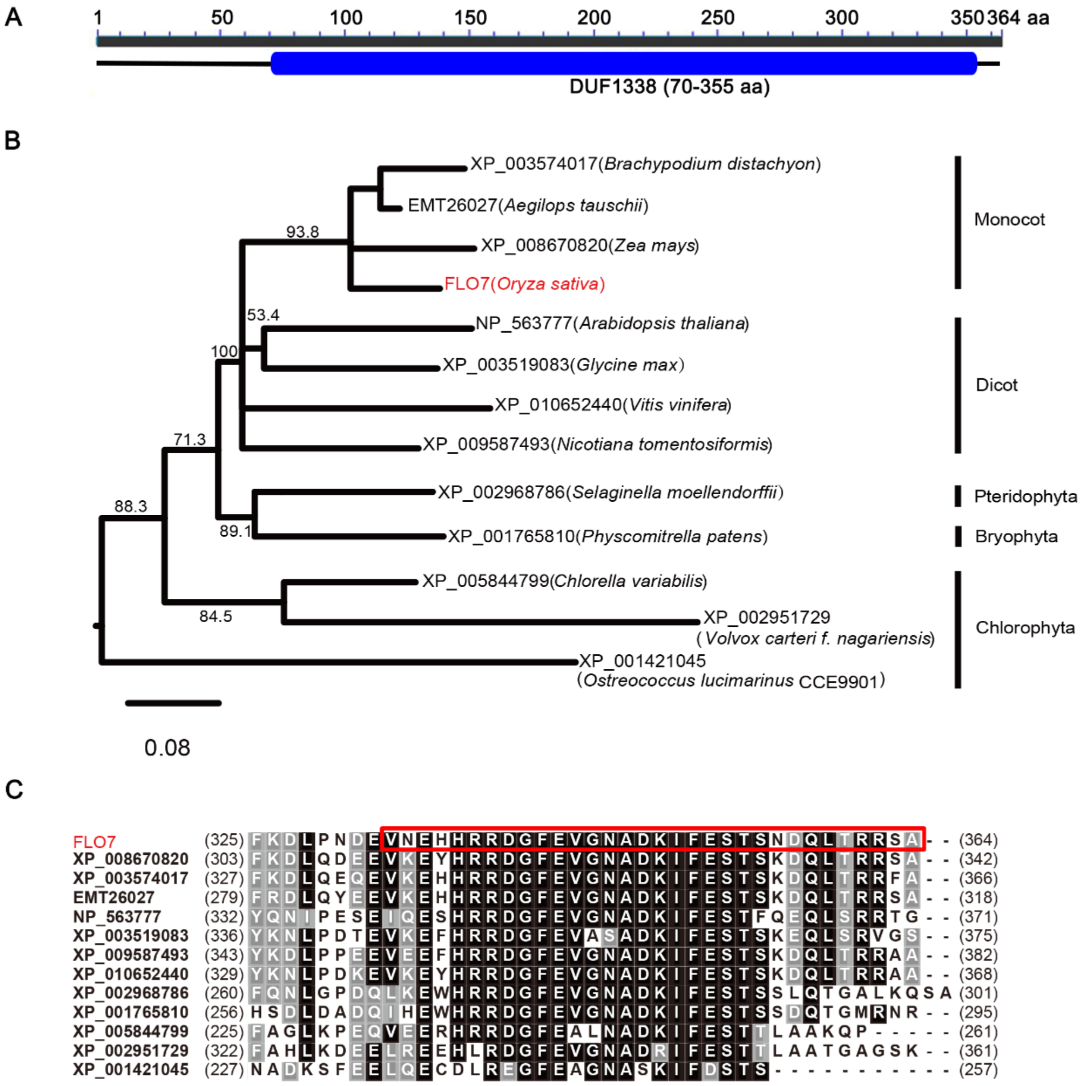

Supplemental Figure S3. Structure and phylogenetic analyses of FLO7 protein.

(A) Schematic domain structure of FLO7. (B) Phylogenetic analysis of FLO7 protein. The tree was derived from alignment of full-length amino acid sequences of FLO7 and its homologous genes from green algae to higher plants. FLO7 (*Oryza sativa*), XP_003574017 (*Brachypodium distachyon*), XP_008670820 (*Zea mays*) and EMT26027 (*Aegilops tauschii*) are representative proteins from monocot plants; NP_563777 (*Arabidopsis thaliana*), XP_003519083 (*Glycine max*), XP_010652440 (*Vitis vinifera*) and XP_009587493 (*Nicotiana tomentosiformis*) are representative proteins from dicot plants. XP_0029668786 (*Selaginellamoellendorffii*) belongs to pteridophyte; XP_001765810 (*Physcomitrella patens*) is from a bryophyte.

XP_005844799 (*Chlorella variabilis*), XP_001421045 (*Volvox carteri f.nagariensis*) and XP_001421045 (*Ostreococcus lucimarinus* CCE9901) are from chlorophyta. All proteins are named according to their gene/EST names or NCBI accession numbers. (C) Multiple amino acid sequence alignments of the C-terminal region of FLO7 proteins. Amino acid sequences were deduced from the orthologs of FLO7 and aligned using ClustalX software. Highly conserved residues are highlighted with black backgrounds. The FLO7 amino acid sequences in the red box indicate the deleted in *flo7* mutant protein.

MAVALAGARSPGAGAILSLRRLAPAAAAPVRLGGSGTPGTRRRRGIAMAAA
ASAPPAPADALPKGADSFFRTVISNMEKVYLSRNPTAKTILELVRSYDGDHI
CYDHFAFRTFGVDGYGIKSLAEFFTDFGYVPREELRFPAKKLRALWFSPPT
NDGYTGTGVYGPLPRIFISELLVDELSPQSQDIIQKYIRTSGKGNKHATLAST
SGELTWEKPIYSDFQVLSRESEYAAWTLVNGYALNHTTISTHRLISDIRSINK
FNKFVEDNGFKLNSEGGILKVSPDGLLQQSSTVADSALFTFADGITESIPRSY
IEFAERLVLPQFKDLPNDEVNEHHRRDGFEVGNADKIFESTSNDQLTRRSA

Supplemental Figure S4. Polypeptide sequences for FLO7 polyclonal antibodies production. Polypeptide sequences for polyclonal antibodies FLO7-C and FLO7-N generation are noted with blue font and red font, respectively. Amino acids in red box indicate the missing polypeptide sequence in *flo7* protein.

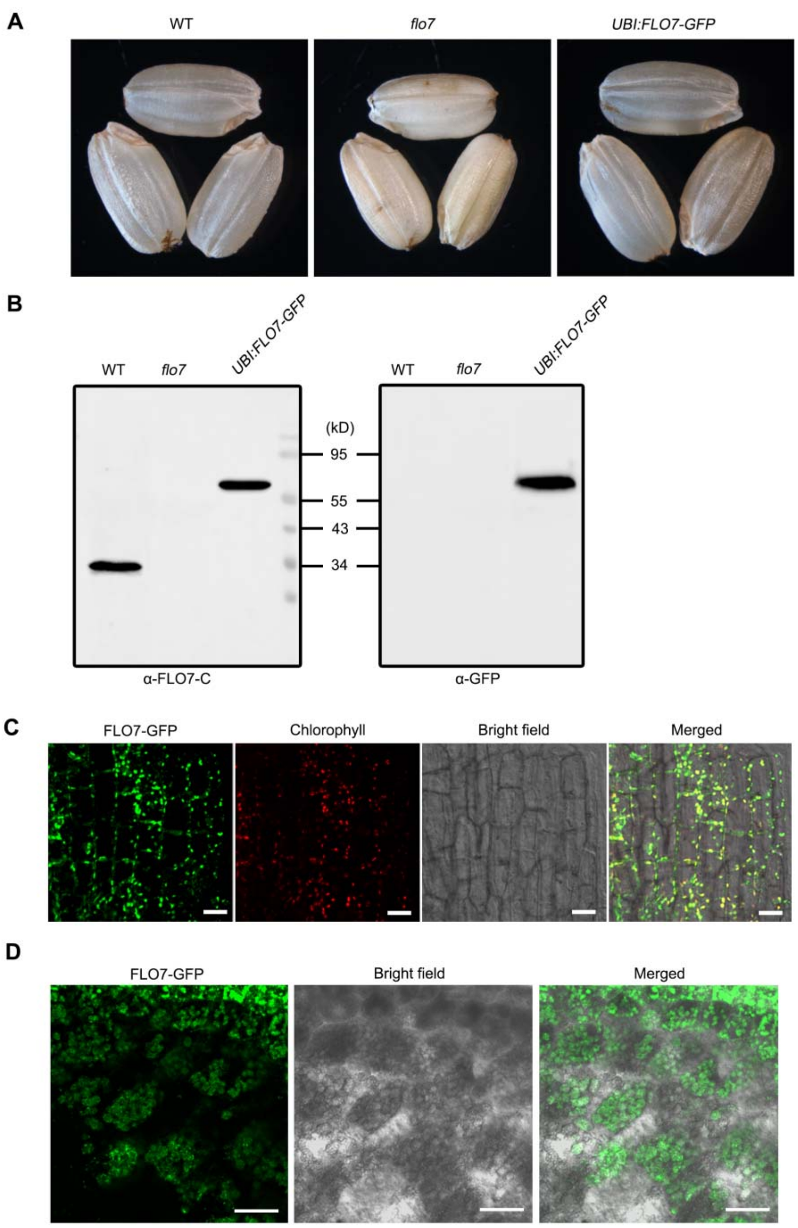

Supplemental Figure S5. Complementation of the *flo7* mutant phenotypes by *UBIQUITIN1* promoter-driven FLO7-GFP.

(A) *UBIQUITIN1* promoter-driven *FLO7-GFP* transgene rescued the grain phenotype of *flo7* mutant. (B) Western blot analysis showing that FLO7-C antibodies can specially detect the endogenous FLO7 and FLO7-GFP fusion proteins from transgenic fragment-positive lines. (C) Chloroplast localization of FLO7 proteins in leaf sheath cells from 5-day-old *FLO7-GFP* transgenic plants. Bars = 20 μm. (D) Amyloplast localizations of FLO7-GFP in the periphery of the complemented transgenic *flo7* endosperm. Bars = 50 μm.

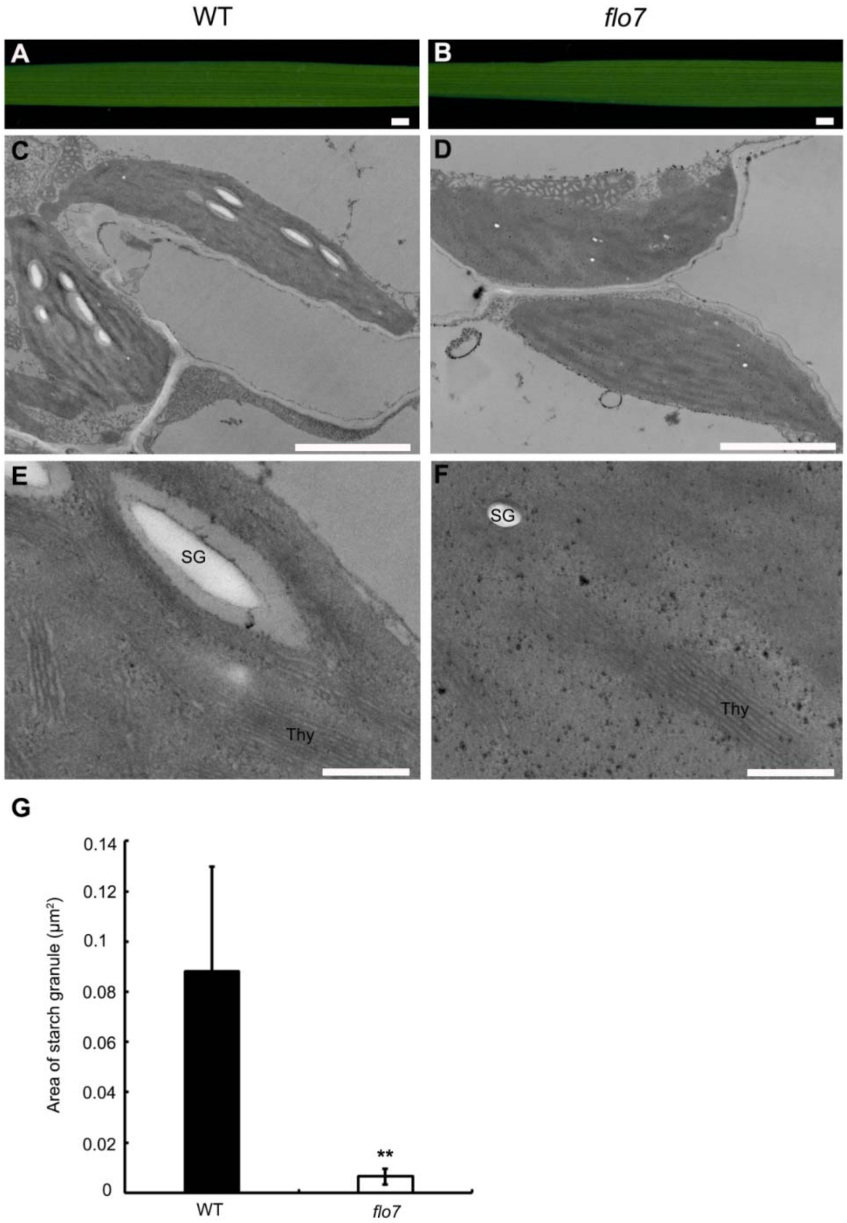

Supplemental Figure S6. Ultrastructure of chloroplasts in mesophyll cells of 2-week-old wild-type and *flo7* seedlings. (A, B) Third leaves from 2-week-old wild-type and *flo7* seedlings, respectively. Bars = 1 mm. (C, D) TEM images of chloroplasts of wild type and *flo7*, respectively. Bars = 2 μm. (E, F) Magnified TEM images showing the SG as well as thylakoid and envelope membrane. SG, starch granule, Thy, thylakoid and envelope membrane. Bars = 200 μm. (G) Quantitative comparison of starch granule areas in wild-type and *flo7* chloroplasts (n = 35 each). Data are given as means ± SD (from at least 3 independent samples) and are compared with wild type by Student's t test (**$P < 0.01$).

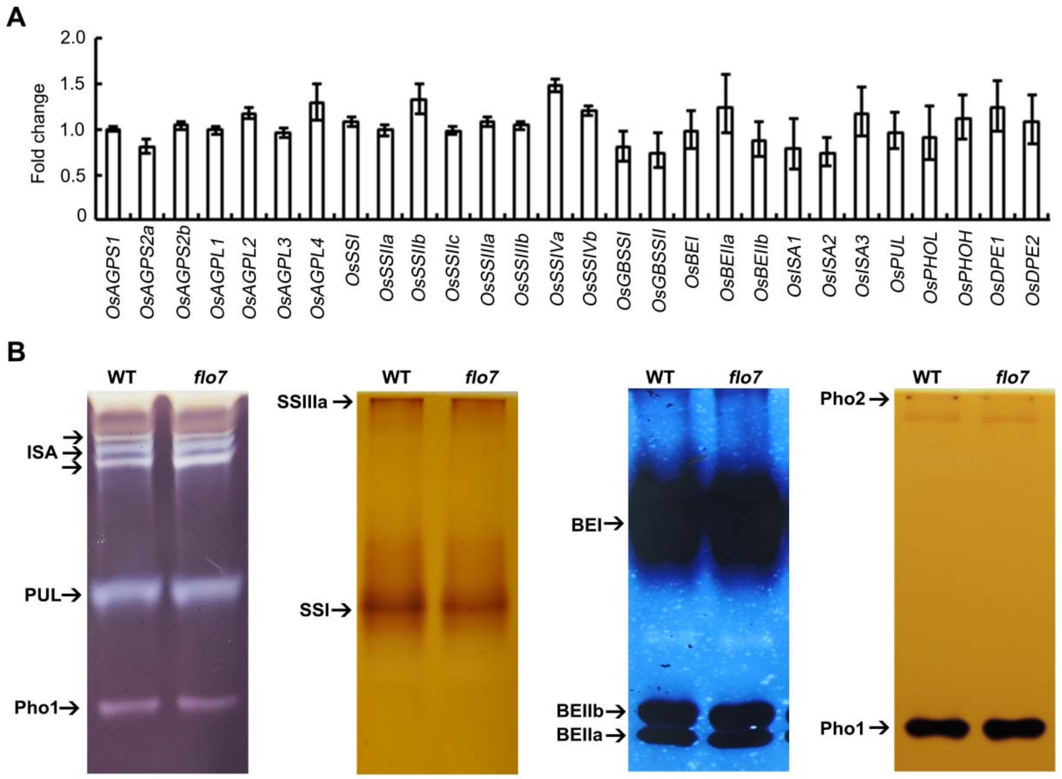

Supplemental Figure S7. Expression levels of the genes involved in production of storage starch as well as zymogram analysis of starch synthesizing enzymes.

(A) Expression Levels of the Genes involved in production of starch synthesis. (B) Zymogram analysis of starch synthesis enzymes.

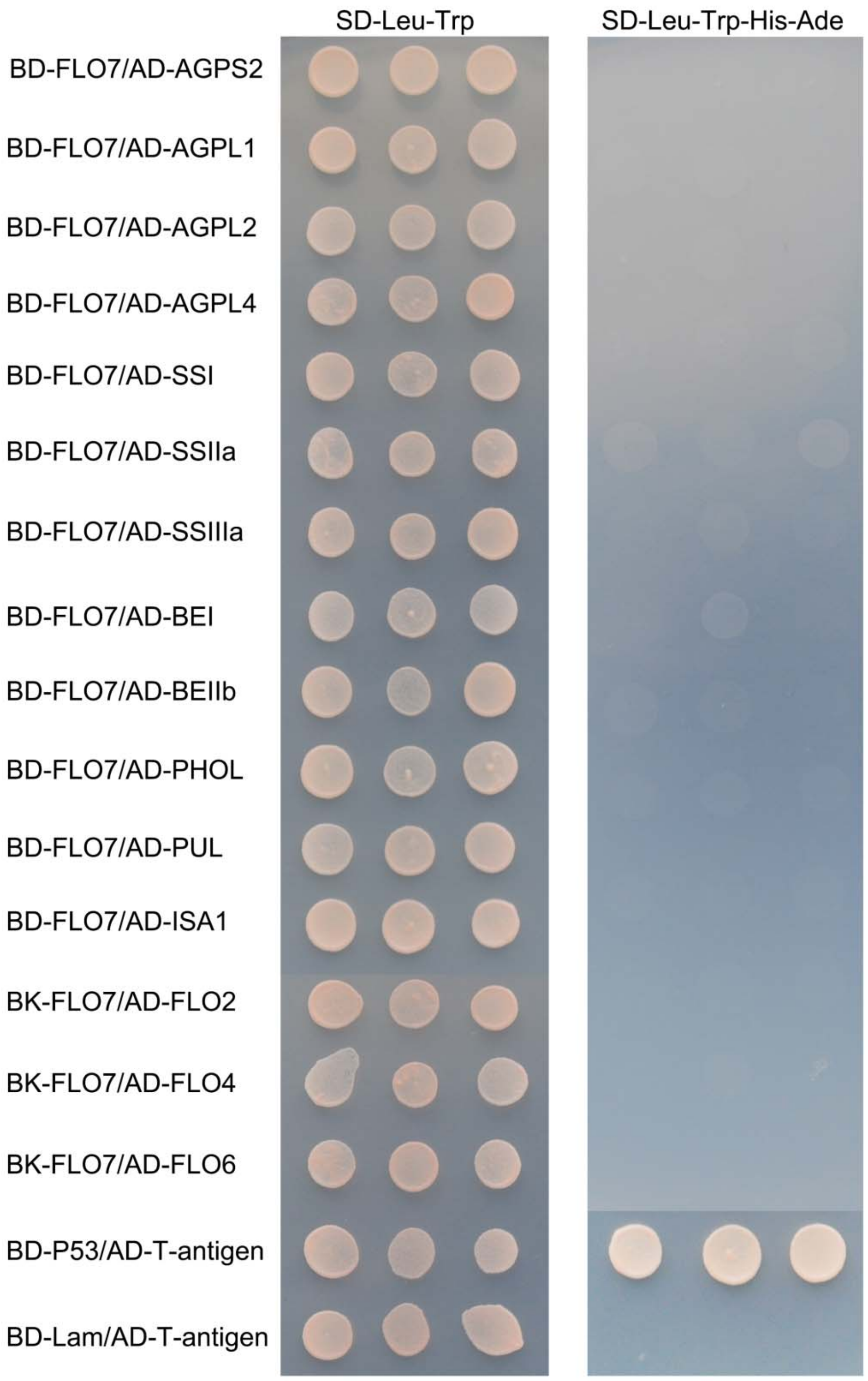

Supplemental Figure S8. Yeast two-hybrid assays between FLO7 and proteins involved in starch synthesis. Yeast two-hybrid assay showing that FLO7 did not interact with starch synthesis-related genes. BD-P53/AD-T-antigen was used as a positive control, while BD-Lam/AD-T-antigen was used as a negative control.

Table S1. Primers used in this study.

Usage	Primer Name	Sequence (5' to 3')
Fine mapping	RM222-F	CTTAAATGGGCCACATGCG
	RM222-R	CAAAGCTTCCGGCCAAAAG
	RM271-F	TCAGATCTACAATTCCATCC
	RM271-R	TCGGTGAGACCTAGAGAGCC
	Z1-F	AATGACAAGGCCGACGATAG
	Z1-R	TATTACCCAGGCCAACCTGT
	Z3-F	CTCAGTTGTTGGGGGATGAG
	Z3-R	CTTTGGAGATGTGCCAGAGA
	Z5-F	TTGATTACAACCAACTCTGACC
	Z5-R	CAATTGAGTAAGTCTGCTTGTGA
	Z7-F	GCGAGTGACAAAGGAGCA
	Z7-R	CCACCACTGTTGTCCCTAAT
	Z10-F	CATCTGCGGGTTGAGTGT
	Z10-R	TGACATTTCAGTTCTATTGGAC
	Z15-F	TTTTGATGATGCCTACTCCA
	Z15-R	GTAATTTGAAAAGCGTGCC
	Z24-F	GCGAGTGACAAAGGAGCA
	Z24-R	CCACCACTGTTGTCCCTAAT
Genotyping	InDel-F	TGGTACTCCCACAGTTCAAAG
	InDel-R	AGAGAACACTCCTTCAATACGC
	2300K-F	TTAATAACACATTGCGGACGT
	2300K-R	GGATTGCACGCAGGTTCTC
Binary vector construction	FLO7- 2300-Sma1-F	GTAGAAGAGGTACCCGGGTTCGCCTCCAGTGCTCGC
	FLO7- 2300 -Sma1-R	CTCTAGAGGATCCCCGGGCACAGAGAACACTCCTTCAATACGC '
	FLO7-p35S-1305-GFP-SpeI-F	GCCCAGATCAACTAGTATGGCCGTGGCCCT
	FLO7- p35S -1305-GFP- BamHI -R	TGCTCACCATGGATCCTGCAGATCTCCGTG
	flo7- p35S -1305-GFP- BamHI -R	TGCTCACCATGGATCCAAACCATCGCGCCTC
	FLO7-pUbi-1305-GFP-SpeI-F	GGTACCTGCAACTAGTATGGCCGTGGCCCTTGCCGGCGC
	FLO7- pUbi -1305-GFP- SpeI -R	CGGACTTAAGACTAGTTGCAGATCTCCGTGTAAGCTGAT
Transient expression assays	PAN580-(1-69aa)-GFP-BamHI -F	CGGTCCCGGGGGATCCATGGCCGTGGCCCTTGCCGG
	PAN580-(1-69aa)-GFP-BamHI -R	TGCTCACCATGGATCCAAATGAATCAGCACCC
	PAN580-(70-364aa)-GFP-BamHI -F	CGGTCCCGGGGGATCCCTTCGGACAGTTATCTCAAACA
	PAN580-(70-364aa)-GFP-BamHI -R	TGCTCACCATGGATCCTGCAGATCTCCGTGTAAGCT
	PAN580-(1-364aa)-GFP-BamHI -F	CGGTCCCGGGGGATCCATGGCCGTGGCCCTTGCCGG
	PAN580-(1-364aa)-GFP-BamHI -R	TGCTCACCATGGATCCTGCAGATCTCCGTGTAAGCT
	PAN580-*flo7*(1-336aa)-GFP-BamHI -F	CGGTCCCGGGGGATCCATGGCCGTGGCCCTTGCCGG
	PAN580-*flo7*(1-336aa)-GFP-BamHI -R	TGCTCACCATGGATCCAAACCATCGCGCCTC
	35S-LUC-NcoI-F	AGATCGAATTCCATGGTCGTACCCCTACTCCAAAAATG
	FLO7-LUC-NcoI-R	TTGGCGTCTTCCATGGCTGCAGATCTCCGTG
	flo7-LUC-NcoI- R	TTGGCGTCTTCCATGGCAAACCATCGCGCCTC

**quantitative RT-PCR**	qRT-FLO7-F	TTATGATGGGGACCACATTTGCTA
	qRT-FLO7-R	AAACCTTAGTTCTTCACGAGGGACA
	qRT-Ubq-F	ACCCTGGCTGACTACAACATC
	qRT-Ubq-R	AGTTGACAGCCCTAGGGTG
**Yeast two-hybrid vector construction**	AD-AGPS1-F	GGAGGCCAGTGAATTCATGGCGATGATGGCGATG
	AD-AGPS1-R	CGAGCTCGATGGATCCTATGACTGTTCCGCTAGGGATT
	AD-AGPL1-F	GGAGGCCAGTGAATTCATGCAGTTCAGCAGT
	AD-AGPL1-R	CGAGCTCGATGGATCCCCTATATGACCTTCCC
	AD-AGPL2-F	GGAGGCCAGTGAATTCATGCAATTCATGATG
	AD-AGPL2-R	CGAGCTCGATGGATCCCTTATATGACGGTCCC
	AD-AGPL4-F	GGAGGCCAGTGAATTCATGGCGACCTGCTCG
	AD-AGPL4-R	CGAGCTCGATGGATCCCCTAGATAACTGTACC
	AD-SSI-F	GGAGGCCAGTGAATTCATGGCGACGGCGGCGGGGAT
	AD-SSI-R	CGAGCTCGATGGATCCTACTGTGAGGCGGCATGGTC
	AD-SSIIa-F	GGAGGCCAGTGAATTCATGTCGTCGGCCGTC
	AD-SSIIa-R	CGAGCTCGATGGATCCCTCACCATTGGTACTT
	AD-SSIIIa-F	GGAGGCCAGTGAATTCATGGATTCATTAGCATTGCA
	AD-SSIIIa-R	CGAGCTCGATGGATCCAAATTTGTGAGCTGAATGG
	AD-BEI-F	GGAGGCCAGTGAATTCATGCTGTGTCTCACC
	AD-BEI-R	CGAGCTCGATGGATCCCTCATTTGCAGTCTTC
	AD-BEIIb-F	GGAGGCCAGTGAATTCATGGCGGCGCCGGC
	AD-BEIIb-R	CGAGCTCGATGGATCCCTCATTCCGCTGGA
	AD-PHOL-F	GGAGGCCAGTGAATTCATGAACTGGAATGCA
	AD-PHOL-R	CGAGCTCGATGGATCCCCTAGGGCAGGATGAC
	AD-PUL-F	GGAGGCCAGTGAATTCATGCAGATGCTGCTC
	AD-PUL-R	CGAGCTCGATGGATCCCTCAACATCTAGGTT
	AD-ISA1-F	GGAGGCCAGTGAATTCATGGCGAGCCTCCCG
	AD-ISA1-R	CGAGCTCGATGGATCCATCATCAGGCTGCA
	AD-FLO2-F	GGAGGCCAGTGAATTCATGGCCCCCAAGGGCGCCGGCC
	AD-FLO2-R	CGAGCTCGATGGATCCGCTGACAGAAGTGCAAATTGACT
	AD-FLO4-F	GGAGGCCAGTGAATTCATGCCGTCGGTTTCGAGGG
	AD-FLO4-R	CGAGCTCGATGGATCCGAGGAGCACCTGAGCTGCAG
	AD-FLO6-F	GGAGGCCAGTGAATTCATGCTCCCCCTCCTCCTCCC
	AD-FLO6-R	CGAGCTCGATGGATCCAGTGACAGTCAGAAGGTTGTT
	BK-FLO7-F	CATGGAGGCCGAATTCATGGCCGTGGCCCTTGCCGG
	BK-FLO7-R	TAGTTATGCGGCCGCTGCAGGTTATGCAGATCTCCGTGTAAG
***In situ* hybridization**	pGEM-FLO7-F	TCAGAGCCAGGACATCATTC
	pGEM-FLO7-R	GGAGACCATCAGGACTCACT
